# Overexpression of ATase1 and ATase2 disrupts the secretome and causes a progeria phenotype

**DOI:** 10.26508/lsa.202503378

**Published:** 2025-09-10

**Authors:** Tzu-Lin Cheng, Feixuan Wu, Md Ezazul Haque, Abigail R Thiel, Danqing Wang, Jeffrey J Helgager, Lingjun Li, Luigi Puglielli

**Affiliations:** 1 Department of Medicine, University of Wisconsin-Madison , Madison, WI, USA; 2 Waisman Center, University of Wisconsin-Madison , Madison, WI, USA; 3 Neuroscience Training Program, University of Wisconsin-Madison , Madison, WI, USA; 4 School of Pharmacy, University of Wisconsin-Madison , Madison, WI, USA; 5 Department of Pathology and Laboratory Medicine, University of Wisconsin-Madison , Madison, WI, USA; 6 Department of Chemistry, University of Wisconsin-Madison , Madison, WI, USA; 7 Geriatric Research Education Clinical Center, Veterans Affairs Medical Center, Madison, WI, USA; 8 Department of Neuroscience, University of Wisconsin-Madison , Madison, WI, USA

## Abstract

Nε-lysine acetylation in the lumen of the ER requires two acetyltransferases, ATase1/NAT8B and ATase2/NAT8. They are type II membrane proteins and belong to the larger GNAT superfamily of acetyltransferases. Their enzymatic activity is tightly coupled to the import of acetyl-CoA in the lumen of the ER by AT-1/SLC33A1. Gene duplication events involving 3q25.31 (harboring *AT-1/SLC33A1*) and 2p13.1 (harboring *ATase1/NAT8B* and *ATase2/NAT8*) are associated with autism spectrum disorder with intellectual disability and progeria-like dysmorphism. Here, we report the generation and phenotypic characterization of mice with systemic overexpression of ATase1 (ATase1 sTg) and ATase2 (ATase2 sTg). Overexpression of either ATase at conception was found to be lethal while overexpression at birth was found to cause a progeria-like phenotype that included skin alterations, lordokyphosis, reduced bone density, sarcopenia, splenomegaly, adenomegaly, and systemic inflammation. The phenotype of ATase1 sTg mice displayed incomplete penetrance, while the phenotype of ATase2 sTg displayed full penetrance and was more severe. Mechanistically, the phenotype was linked to altered dynamics of the secretory pathway with defects affecting the quality of the secretome.

## Abstract (precis)

This study shows that overexpression of the acetyltransferases ATase1 and ATase2 in the mouse alters the quality of the secretory pathway causing a progeria-like phenotype.

## Graphical Abstract

**Figure:**
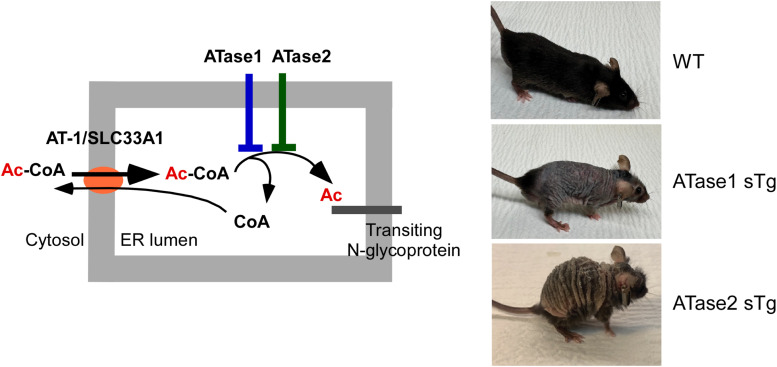

## Introduction

Nε-lysine acetylation is a highly dynamic post-translational event that regulates a variety of protein functions (Puglielli et al, 2023). In the lumen of the ER, Nε-lysine acetylation is carried out by ATase1/NAT8B and ATase2/NAT8 (Fernandez-Fuente et al, 2023b). Both ATases are type II membrane proteins; the catalytic domain is exposed to the lumen of the organelle and displays the conserved R/Q-x-x-G-x-G/A acetyl-CoA binding motif of the GNAT superfamily of Nε-lysine acetyltransferases (Fernandez-Fuente et al, 2023b). The activity of the ATases is tightly coupled to the influx of acetyl-CoA from the cytosol, which is ensured by AT-1/SLC33A1, an ER membrane transporter that acts as an antiporter by coupling the cytosol-to-ER transfer of acetyl-CoA with the ER-to-cytosol exit of free CoA (Fernandez-Fuente et al, 2023b).

Defective ER acetylation, as caused by either mutations or gene duplication events, is associated with severe rare human diseases (Fernandez-Fuente et al, 2023b). Importantly, duplications involving 3q25.31 (harboring *AT-1/SLC33A1*) and 2p13.1 (harboring *ATase1/NAT8B* and *ATase2/NAT8*) are specifically associated with autism spectrum disorder with intellectual disability and progeria-like dysmorphism (see National Organization for Rare Disorders database; see also [Francke, 1978; Fineman et al, 1983; Fryns et al, 1989; Sawyer et al, 1994; Rizzu et al, 1997; Ounap et al, 2005; Krumm et al, 2013; Poultney et al, 2013; Krumm et al, 2015]). In the case of AT-1, the disease association was successfully modeled (and validated) in the mouse. Indeed, mice knock-in for a loss-of-function AT-1 mutation associated with spastic paraplegia developed a peripheral form of neuropathy (Peng et al, 2014; Liu et al, 2017), mice with neuron-specific overexpression of AT-1 developed an autistic-like phenotype (Hullinger et al, 2016), and mice with systemic overexpression of AT-1 developed a progeria-like phenotype with reduced lifespan (Peng et al, 2018). However, the disease association with the ATases is yet to be validated in a model organism.

Substrates of ATase1 and ATase2 include both ER-resident and -trafficking proteins (Pehar et al, 2012; Rigby et al, 2021). ER-resident proteins that are modified by the ATases are involved in a variety of functions, including regulation of folding and quality control within the ER lumen, selection of correctly folded proteins to be transported to the Golgi apparatus, and activation of ER-specific autophagy (Farrugia & Puglielli, 2018; Fernandez-Fuente et al, 2023b). In essence, the ATases are predicted to influence functional dynamics of the secretory pathway by regulating protein homeostasis (proteostasis) within the ER/secretory pathway. The implication of the ATases in the regulation of ER-specific authophagy was successfully tested in Atase1^−/−^ and Atase2^−/−^ mice (Rigby et al, 2021). However, the physiological consequences of ATase overexpression are yet to be tested.

Here, we report the generation and phenotypic characterization of mice with systemic overexpression of ATase1 (ATase1 sTg) or ATase2 (ATase2 sTg). Overexpression of either ATase at conception was found to be lethal while overexpression at birth was found to cause a progeria-like phenotype that included skin alterations, lordokyphosis, reduced bone density, sarcopenia, splenomegaly, adenomegaly, and systemic inflammation. Interestingly, the phenotype of ATase1 sTg mice displayed incomplete penetrance and was less severe, while the phenotype of ATase2 sTg displayed full penetrance and was more severe.

Although they share enzymatic activity and can compensate each other in vivo, the ATases are regulated differently, and differently influence the activation of autophagy as well as the global acetyl-CoA metabolic response (Rigby et al, 2020, 2021). Here, we took advantage of these new mouse models and were able to dissect their specific biological roles. Importantly, overexpression of the ATases altered the ER-to-Golgi transition of nascent glycoproteins and forced selective and divergent adaptive responses outside of the ER.

In conclusion, this study highlights specific and fundamental biological functions of ER acetylation and reveals novel adaptive responses to ER-specific Nε-lysine acetylation within the cell that impact the pathophysiology of associated human diseases.

## Results

### ATase1 sTg and ATase2 sTg mice display a progeria-like phenotype

**Figure 1.**
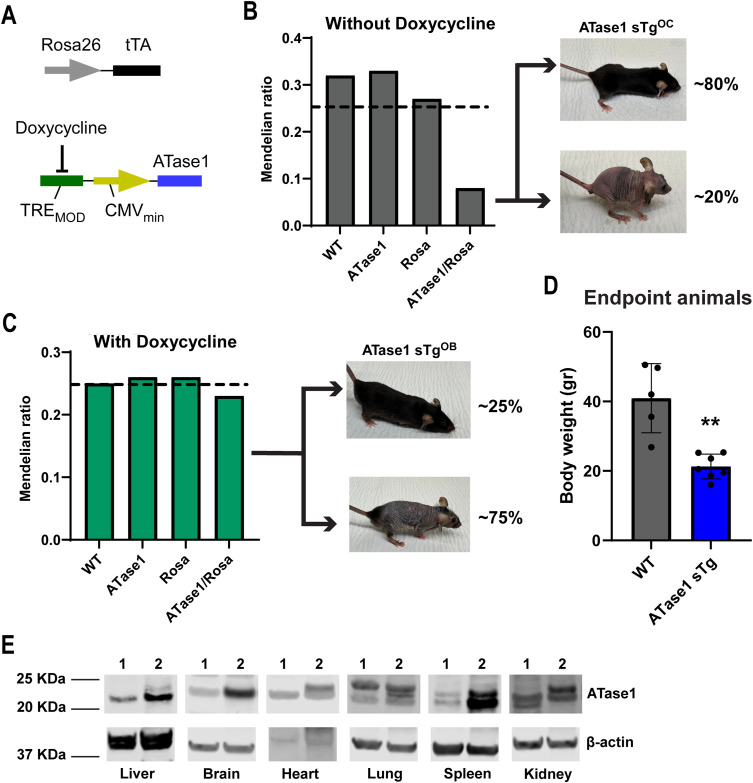
Systemic overexpression of human ATase1 caused a progeria-like phenotype with incomplete penetrance. **(A)** ATase1 sTg mice were generated with a Tet-Off expression system driven by the Rosa26 locus. **(B)** Overexpression of ATase1 from conception reduced the yield of ATase1 sTg mice. The progeria-like phenotype of ATase1 sTg^OC^ mice displayed incomplete penetrance. **(C)** Overexpression of ATase1 at birth restored the Mendelian ratio. The progeria-like phenotype of ATase1 sTg^OB^ mice displayed incomplete penetrance. **(D)** ATase1 sTg mice with progeria-like phenotype displayed reduced body weight. The number of animals is shown. **P < 0.005. Welch’*t* test. **(E)** Western blot showing overexpression of ATase1 in different organs (1. WT mice, 2. ATase1 sTg mice). Mice were ∼1 yr old when studied. Source data are available for this figure.

**Figure 2.**
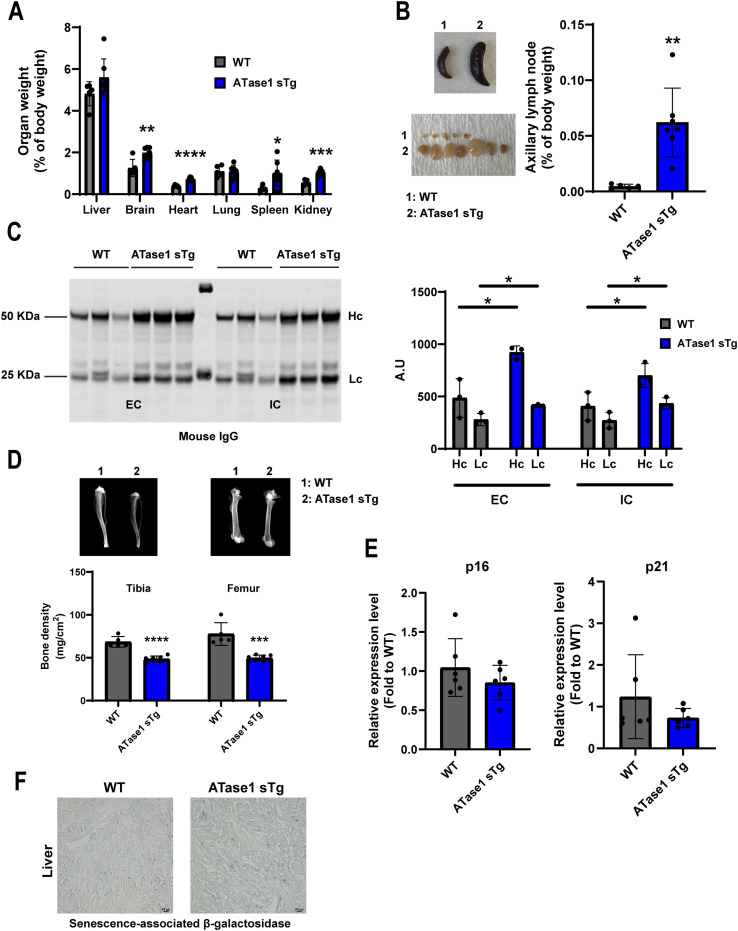
ATase1 sTg mice displayed progeria-like features. **(A)** Organ to body weight ratio. The number of animals is shown. *P < 0.05; **P < 0.005; ***P < 0.0005; ****P < 0.0001. Unpaired *t* test or Welch’*t* test. **(B)** ATase1 sTg mice displayed splenomegaly and adenomegaly (axillary lymph nodes are shown). The number of animals is shown. **P < 0.005. Welch’*t* test. **(C)** ATase1 sTg mice displayed increased Ig tissue infiltration (liver). The left panel shows the Western blot while the right panel shows the associated quantification. The number of animals is shown. *P < 0.05. Unpaired *t* test. EC, extracellular; IC, intracellular; Hc, heavy chain; Lc, light chain. **(D)** ATase1 sTg mice displayed reduced bone density (Tibia and Femur are shown). The number of animals is shown. ***P < 0.0005; ****P < 0.0001. Unpaired *t* test or Welch’*t* test. **(E)** Senescence markers p16 and p21 (brain). **(F)** Senescence associated β-galactosidase staining (liver). Mice were ∼1 yr old when studied. Source data are available for this figure.

To study the systemic effect of ATase1 and ATase2 overexpression, we used an inducible Tet-Off system driven by the Rosa26 locus that can be inactivated with doxycycline (Peng et al, 2018; Fernandez-Fuente et al, 2023a). Overexpression of ATase1 from conception (ATase1 sTg^OC^) yielded less than 10% of double transgenic mice, which is below the expected Mendelian ratio. Of them, about 20% developed a progeria-like phenotype while the remaining 80% displayed no significant disease manifestations throughout our study (Fig 1A and B). Administration of doxycycline to the pregnant females restored the Mendelian ratio, thus confirming the embryonic lethality of ATase1 systemic overexpression (Fig 1C). When ATase1 was overexpressed at birth (ATase1 sTg^OB^), by removing doxycycline from the diet, about 75% of the double transgenic animals developed a progeria-like phenotype with delayed growth that was indistinguishable from the phenotype of ATase1 sTg^OC^ mice (Fig 1B–D). ATase1 sTg^OB^ animals (simply referred to as ATase1 sTg thereafter) were used as the main model for our study. Evidence of systemic overexpression of human ATase1 was documented by Western blot (Fig 1E). At humane endpoint, which occurred around the age of 1 yr, ATase1 sTg mice displayed reduced body weight (Fig 1D), hair loss with skin lesions (Fig 1C), cardiomegaly (Fig 2A), splenomegaly (Fig 2A and B), adenomegaly (Fig 2B), systemic inflammation (Fig 2C), reduced bone density (Fig 2D), rectal prolapse, and modest sarcopenia (Table 1). When we analyzed the levels of the senescent markers p16, p21, and β-Gal, we only observed a modest up-regulation of β-Gal staining (Fig 2E and F).

**Table 1.** Observed phenotype of ATase1 and ATase2 sTg mice

​	ATase1	ATase2
Lifespan	Reduced	Reduced
Endpoint	1 yr	2–3 mo
Body weight	Reduced	Reduced
Hunched posture	Observed	Observed
Hair loss	Observed	Observed
Skin lesion	Observed	Observed
Muscle atrophy	Observed	Observed
Adipose tissue	Reduced	Reduced
Bone density	Reduced	Reduced
Liver size	Normal	Increased
Heart size	Increased	Normal
Spleen size	Increased	Increased
Lymph node size	Increased	Increased
Rectal prolapse	Observed	Observed
Systemic inflammation	Observed	Observed

ATase1 sTg only includes animals with phenotype.

**Figure 3.**
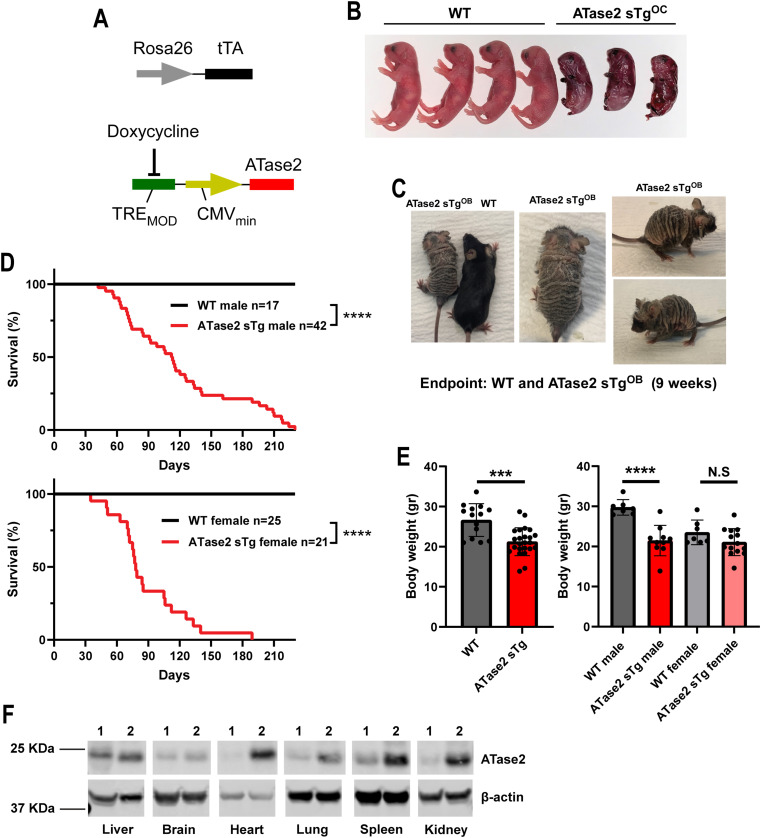
Systemic overexpression of human ATase2 caused a progeria-like phenotype. **(A)** ATase2 sTg mice were generated with a Tet-Off expression system driven by the Rosa26 locus. **(B)** Overexpression of ATase2 from conception was embryonically lethal. Incompletely developed ATase2 sTg^OC^ are shown. **(C)** Overexpression of ATase2 at birth caused a fully penetrant progeria-like phenotype. One representative ATase2 sTg^OB^ and WT littermate are shown next to each other. **(D)** ATase2 sTg mice displayed short life span. Maximum lifespan: males = 230 d, females = 189 d. The number of animals is shown. ****P < 0.0001 via the Kaplan-Meier lifespan test. **(E)** ATase2 sTg mice displayed reduced body weight at humane endpoint. The number of animals is shown. ***P < 0.0005; ****P < 0.0001. Unpaired *t* test. **(F)** Western blot showing overexpression of ATase2 in different organs (1. WT mice, 2. ATase2 sTg mice). Mice were ∼3 mo old when studied. Source data are available for this figure.

Next, we overexpressed human ATase2 using the same Rosa26 system (Fig 3A). As with ATase1 sTg^OC^, systemic overexpression of ATase2 from conception (ATase2 sTg^OC^) was embryonically lethal (Fig 3B). Two main differences were observed between ATase1 sTg^OC^ and ATase2 sTg^OC^. First, the effect of ATase2 overexpression was fully penetrant; no live double transgenic mouse was ever obtained. Second, we recovered incompletely developed dead fetuses from ATase2 sTg^OC^ females but not from ATase1 sTg^OC^ females. This could suggest that the two ATases have partially distinct developmental roles with the overexpression of ATase1 affecting early developmental events, albeit with incomplete penetrance, and overexpression of ATase2 affecting late developmental events.

**Figure 4.**
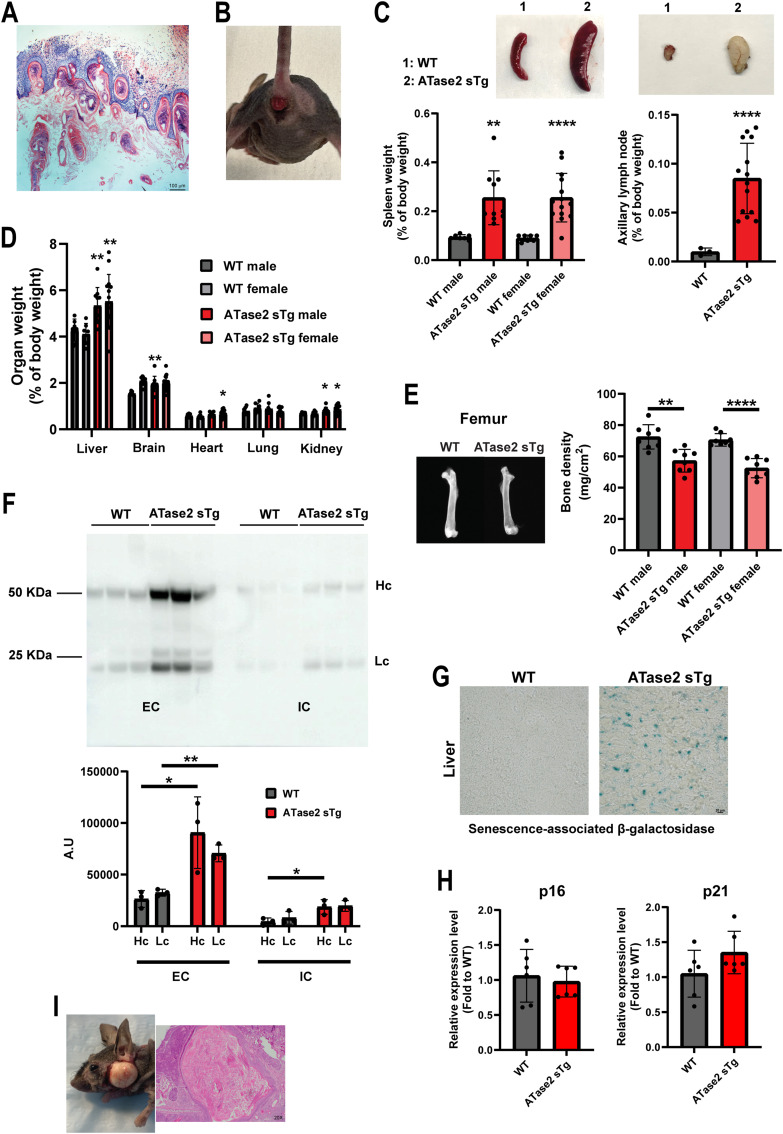
ATase2 sTg mice displayed progeria-like features. **(A)** Representative H&E staining of skin sections of ATase2 sTg mice. **(B)** Rectal prolapse in ATase2 sTg mice. **(C)** ATase2 sTg mice displayed splenomegaly and adenomegaly (axillary lymph nodes are shown). Top panel, representative images. Bottom panel, quantification. The number of animals is shown. **P < 0.005; ****P < 0.0001. Welch’*t* test. **(D)** Organ to body weight ratio. The number of animals is shown. *P < 0.05; **P < 0.005. Unpaired *t* test or Welch’*t* test. **(E)** ATase2 sTg mice displayed reduced bone density (Femur is shown). The number of animals is shown. **P < 0.005; ****P < 0.0001. Unpaired *t* test. **(F)** ATase2 sTg mice displayed increased Ig tissue liver infiltration. The top panel shows the Western blot while the bottom panel shows the associated quantification. The number of animals is shown. *P < 0.05; **P < 0.005. Unpaired *t* test. EC, extracellular; IC, intracellular; Hc, heavy chain; Lc, light chain. **(G)** Senescence associated β-galactosidase staining (liver). **(H)** Senescence markers p16 and p21 (brain). **(I)** Representative gross photograph and H&E-stained section of tumor-like epidermal inclusion cyst in one ATase2 sTg mouse. Mice were ∼3 mo old when studied. Source data are available for this figure.

When ATase2 was overexpressed at birth (ATase2 sTg^OB^), by removing doxycycline from the diet, all the double transgenic animals developed a progeria-like phenotype (Fig 3C). ATase2 sTg^OB^ animals (simply referred to as ATase2 sTg thereafter) were used as the main model for our study. Within a few weeks, ATase2 sTg mice developed hair loss and skin lesions, and by the age of ∼2 mo they all manifested a severe progeria-like phenotype (Fig 3C). The animals also displayed growth delay and a short lifespan (Fig 3C–E). Evidence of systemic overexpression of human ATase2 was documented by Western blot (Fig 3F). The phenotype of ATase2 sTg mice included histological alterations of the skin (Fig 4A), rectal prolapse (Fig 4B), splenomegaly (Fig 4C), adenomegaly (Fig 4C), hepatomegaly (Fig 4D), reduced bone density (Fig 4E), sarcopenia, systemic inflammation (Fig 4F), and up-regulation of β-Gal staining (Fig 4G), which was observed in the absence of increased p16 and p21 markers (Fig 4H; see also Table 1). Finally, about 40% of ATase2 sTg mice developed tumor-like lesions histologically compatible with epidermal inclusion cysts in the area corresponding to the parotid and submandibular gland region (Fig 4I).

In conclusion, systemic overexpression of either ATase resulted in a phenotype that was reminiscent of segmental forms of progerias (Table 1) (Pivnick et al, 2000; Liao & Kennedy, 2014; Gonzalo et al, 2017; Karikkineth et al, 2017). When compared with ATase1 sTg mice, the phenotype of ATase2 sTg mice was fully penetrant and always more severe.

### ATase1 sTg and ATase2 sTg mice display widespread proteomic changes

**Figure 5.**
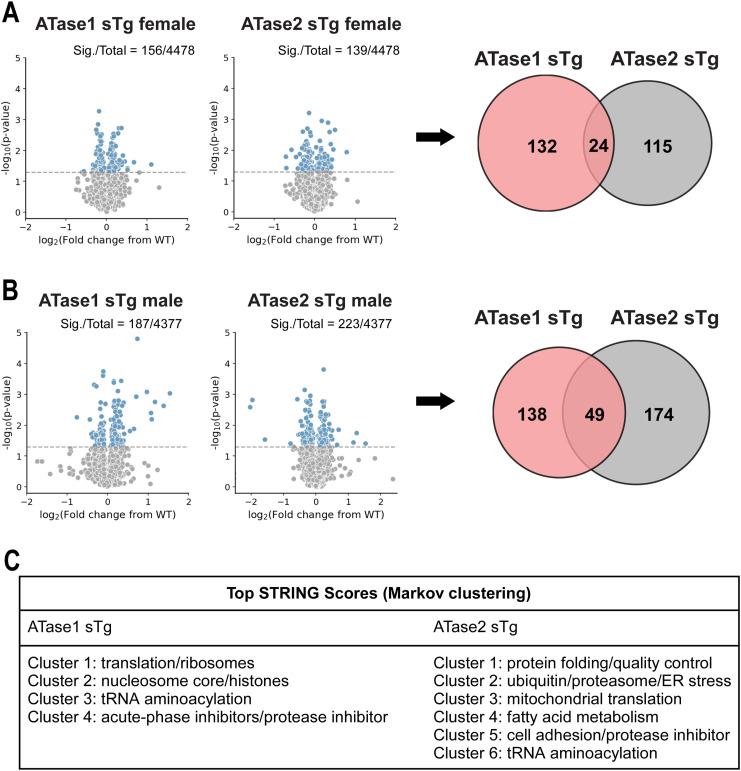
ATase1 sTg and ATase2 sTg mice displayed significant proteomic changes with minor overlap among models. **(A)** Left panel, volcano plot of ATase1 and ATase2 sTg female mice. Significantly changed proteins are shown in blue. Right panel, Venn diagram with overlapping proteins among models. Only significantly altered proteins are shown. N = 4 mice/group. **(B)** Left panel, volcano plot of ATase1 and ATase2 sTg male mice. Significantly changed proteins are shown in blue. Right panel, Venn diagram with overlapping proteins among models. Only significantly altered proteins are shown. N = 4 mice/group. **(C)** Top STRING protein clusters in ATase1 sTg and ATase2 sTg mice. Mice were ∼3 mo old when studied.

**Figure S1.**
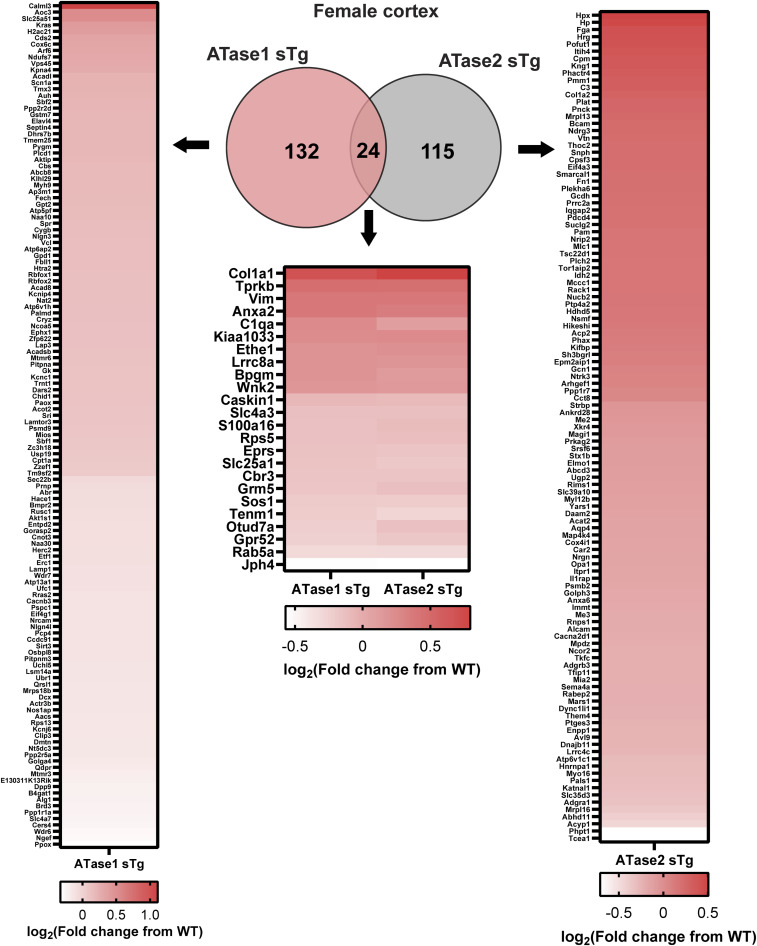
Venn diagram and heatmaps of significantly changed proteins in female ATase1 sTg and ATase2 sTg mouse cortex.

**Figure S2.**
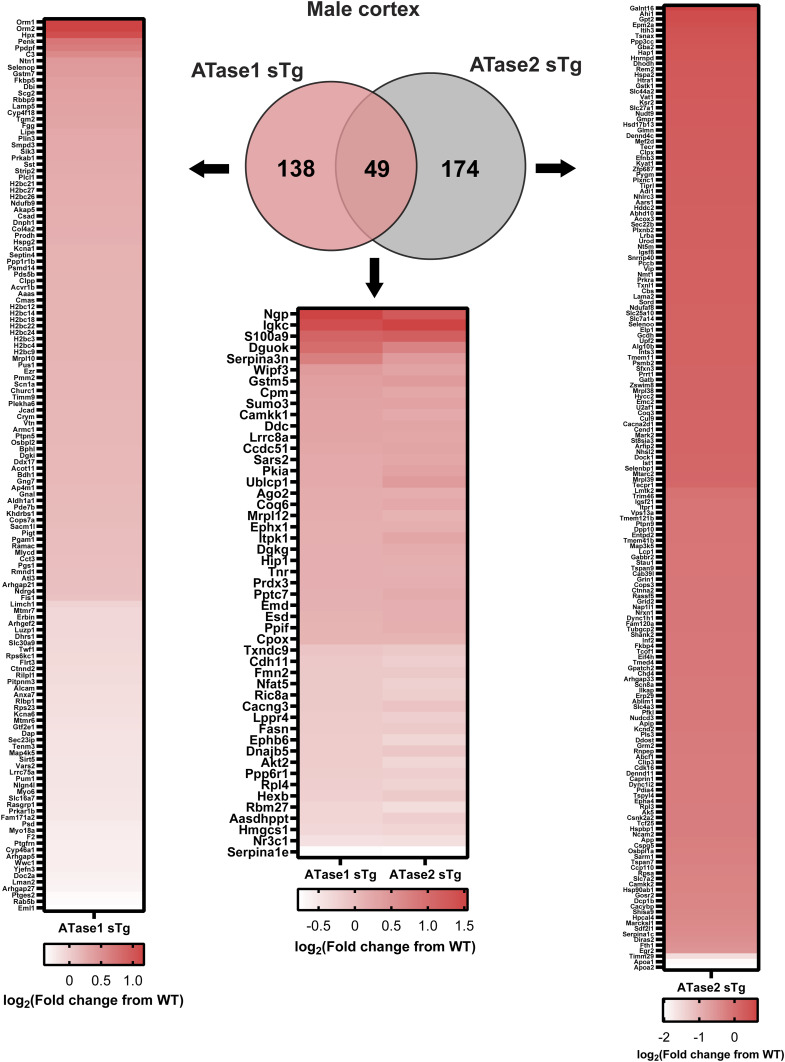
Venn diagram and heatmaps of significantly changed proteins in male ATase1 sTg and ATase2 sTg mouse cortex.

**Figure S3.**
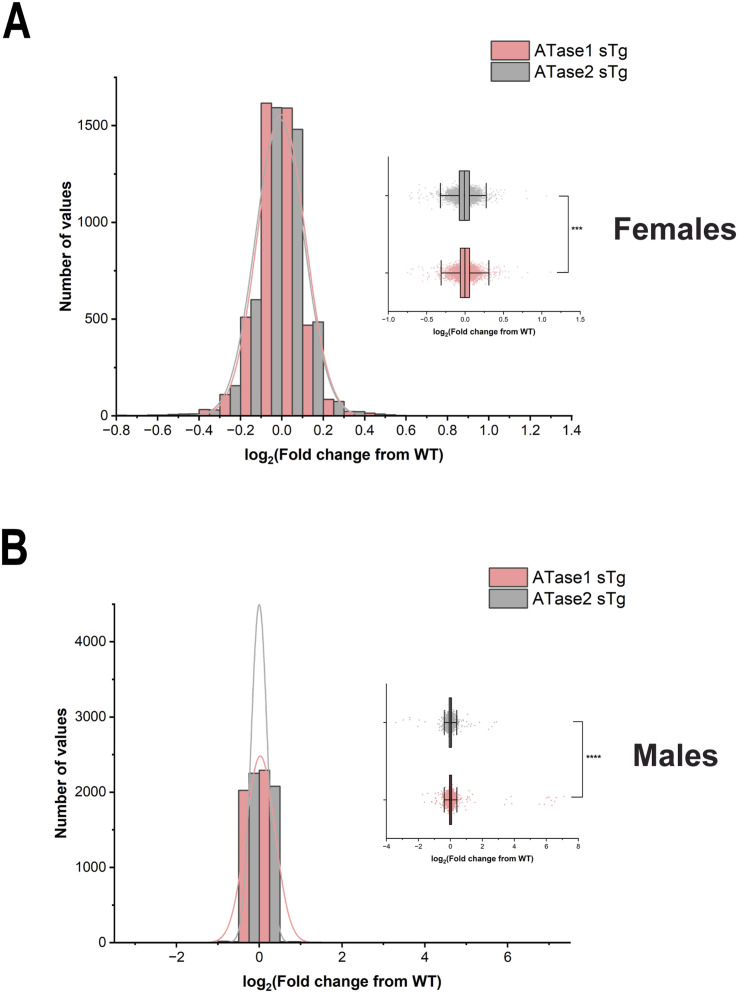
**(A, B)** Histogram and overlaid Gaussian distribution of the female (A) and male (B) ATase1 sTg and ATase2 sTg mouse cortex proteome.

To elucidate the impact of increased ATase activity on protein expression, we conducted large-scale liquid chromatography coupled to tandem mass spectrometry (LC-MS/MS) analysis to identify global changes in the proteome of ATase1 sTg and ATase2 sTg animals. Since the ER acetylation machinery is heavily involved in dynamics of the secretory pathway (Fernandez-Fuente et al, 2023b), we performed our study with brain cortical and hippocampal tissue. In the cortex, we found 156 and 139 proteins being altered in ATase1 and ATase2 sTg females, respectively (Fig 5A). The profile was quite different among the two models, with only 24 proteins being represented in both (Figs 5A and S1). A similar outcome was observed when we compared the males. Indeed, 187 and 223 proteins, respectively, were found altered with only 49 being represented in both models (Figs 5B and S2). In essence, overexpression of either ATase yielded widespread but significantly different proteomic changes in the brain of the two models (Fig S3). The list of significantly changed proteins in the cortex across models with relevant heatmaps is provided in Figs S1 and S2. STRING analysis at >90% confidence revealed four clusters in ATase1 sTg and six clusters in ATase2 sTg that correlated highly with specific cellular process (Fig 5C). They were translation/ribosomes, nucleosome core/histones, tRNA aminoacylation and acute-phase inhibitors/protease inhibitors in ATase1 sTg mice; and protein folding/quality control, ubiquitin/proteasome/ER stress, mitochondrial translation, fatty acid metabolism, cell adhesion/protease inhibitors, and tRNA aminoacylation in ATase2 sTg mice (Fig 5C). Clusters 1 (translation and ribosomes) and 3 (tRNA aminoacylation) in ATase1 sTg, and clusters 1 (protein folding and quality control) and 6 (tRNA aminoacylation) in ATase2 sTg mice are highly relevant to protein biosynthesis and regulation of the secretory pathway, a function that has been connected to the ER acetylation machinery. Cluster 2 (ubiquitin/proteasome/ER stress) in ATase2 sTg included proteins that are relevant to the ER-associated degradation system. Finally, both models displayed general metabolic outcomes, as highlighted by cluster 2 (nucleosome core/histones) in ATase1 sTg and clusters 3 (mitochondrial translation) and 4 (fatty acid metabolism) in ATase2 sTg mice (Fig 5C).

### ATase1 sTg and ATase2 sTg mice display widespread changes in the N-glycosylation profile of transiting proteins

**Figure 6.**
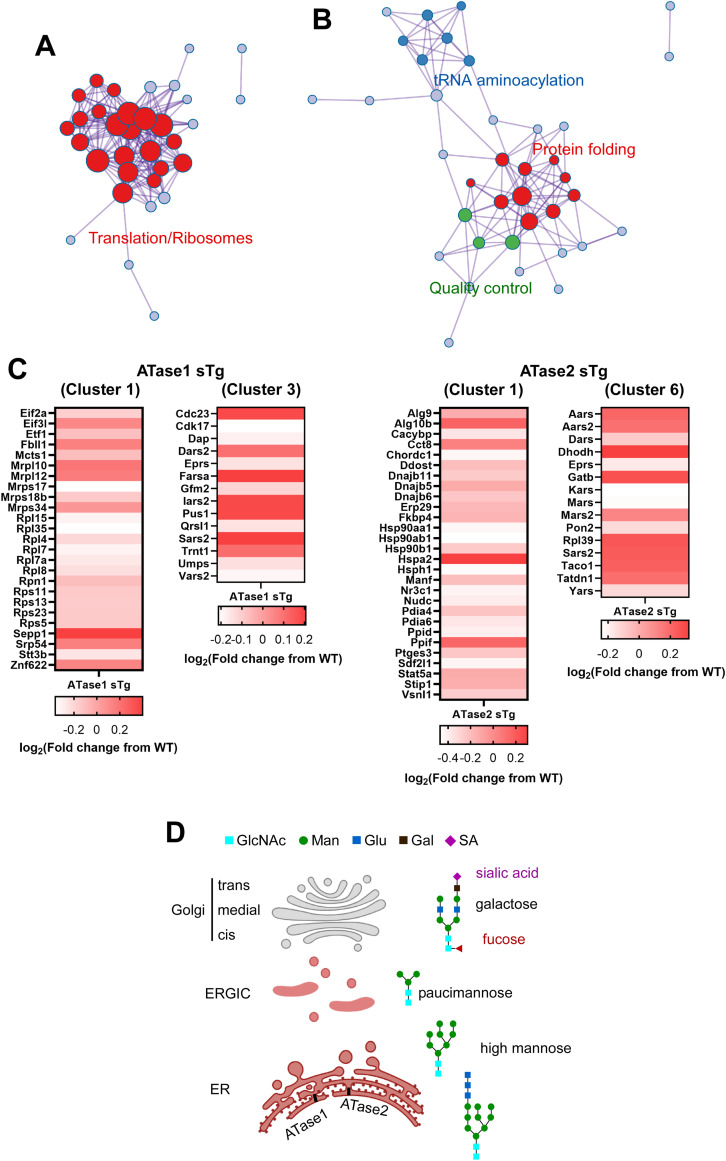
ATase1 sTg and ATase2 sTg mice displayed widespread changes among the protein biosynthetic pathway. **(A, B)** Ontology enrichment of clusters 1 and 3 from ATase1 sTg (A) and clusters 1 and 6 from ATase2 sTg mice (B). Cluster labeling is shown in Fig 5C. **(C)** Heatmaps of clusters 1 and 3 from ATase1 sTg and cluster 1 and 6 from ATase2 sTg mice. Cluster labeling is shown in Fig 5C. **(D)** Schematic diagram of N-glycosylation across the secretory pathway. Mice were ∼3 mo old when studied.

The largest proteomic changes in both sTg models were found to be relevant for protein biosynthesis and regulation of the secretory pathway subgroups (Fig 6A–C). They included translation initiation factors, ribosomal associated proteins, enzymes necessary for folding, chaperones and quality control-associated proteins, as well as integral components of the oligosaccharyl transferase (OST) complex, which is responsible for the initial N-glycosylation of nascent glycoproteins within the ER lumen. Taken together, these data suggest that the ability of the ER to control the engagement of the secretory pathway and the quality of the *secretome* might be affected in the sTg animals.

Within the conventional secretory pathway, newly synthesized proteins that survive quality control in the ER translocate to the Golgi apparatus to complete post-translational maturation and then be delivered to their final destination. Importantly, N-glycosylation begins in the lumen of the ER where the OST transfers a pre-formed GlcNAc_2_Man_9_Glc_3_ oligosaccharide structure onto an Asn residue within the N-X-S/T consensus motif (Fig 6D). This initial oligosaccharide undergoes major modifications as glycoproteins move out of the ER and through the Golgi apparatus. Specifically, the three terminal glucose are removed and the high-mannose structure is trimmed to allow final modification, which includes addition of fucose and galactose in the *cis/medial*-Golgi and sialic acid in the *trans*-Golgi and *trans*-Golgi network (Fig 6D) (Hirschberg et al, 1998). In essence, the structure of the oligosaccharide complex reflects the ability of the ER to deliver correctly folded N-glycoproteins to the Golgi apparatus as well as the ability of the Golgi apparatus to modify transiting N-glycoproteins.

Importantly, we previously reported that the ATases interact with the OST within a high-molecular mass complex suggesting an intimate connection between OST-dependent N-glycosylation and ATase-dependent Nε-lysine acetylation of newly synthesized N-glycoproteins (Ding et al, 2014). This argument is reinforced by the dataset included here (Fig 6C), as reflected by changes in the levels of Stt3b, Rpn1, and Ddost, which are integral components of the mouse Ost super-complex, as well as Alg9 and Alg10b, which are involved in the assembly of the initial GlcNAc_2_Man_9_Glc_3_ oligosaccharide structure. Therefore, to determine whether the changes reflected by the proteome data impinge on the quality of the *secretome* by influencing the ER-to-Golgi transition of nascent proteins, we used sequential hydrophilic interaction chromatography (HILIC) to enrich intact N-glycopeptides and LC-MS/MS to analyze the composition of the oligosaccharides and the incorporation of Golgi-specific sugars. To be consistent with the design of the proteome, we characterized the glycoproteome of the brain.

**Figure 7.**
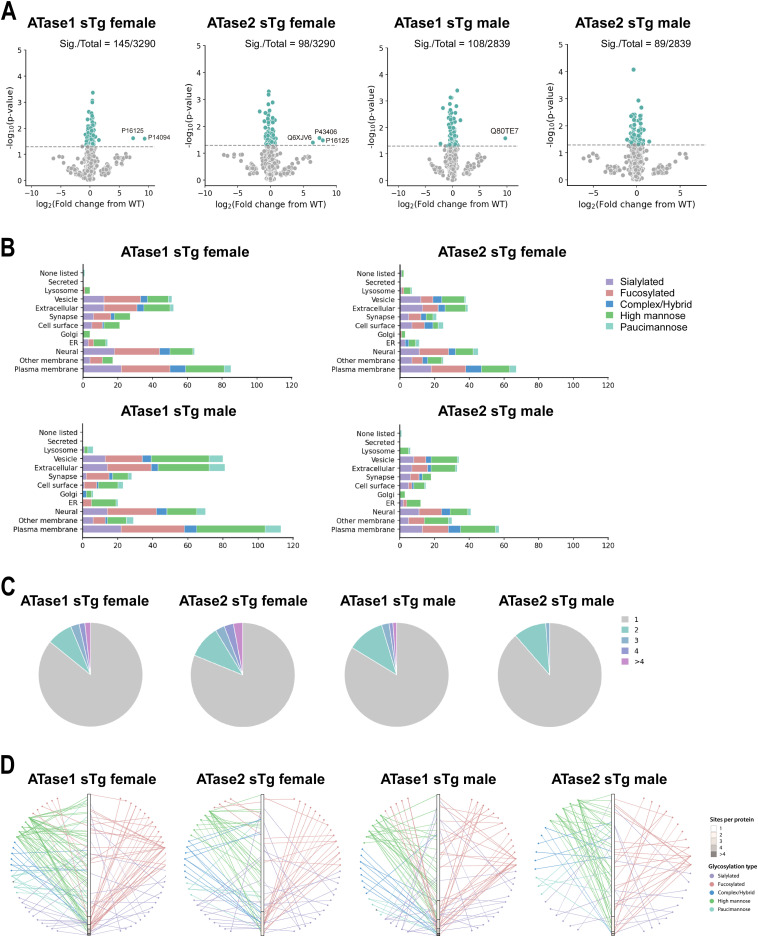
ATase1 sTg and ATase2 sTg mice displayed significant alterations within the N-glycoproteome (cortex). **(A)** Volcano plots showing quantified glycoproteins. Statistically significant glycoproteins are shown in green. N = 4 mice/group. Significance was calculated at *P* < 0.05 via *t* test. **(B)** Glycosylation distribution across different subcellular localizations as defined by GO cellular component terms. Only significantly altered glycans are shown. **(C)** Pie charts showing the distribution of altered glycans per glycosite. Only statistically significant glycans are shown. **(D)** Association network of the type and number of glycans per glycoprotein. Only statistically significant glycans are shown. Mice were ∼3 mo old when studied.

**Figure S4.**
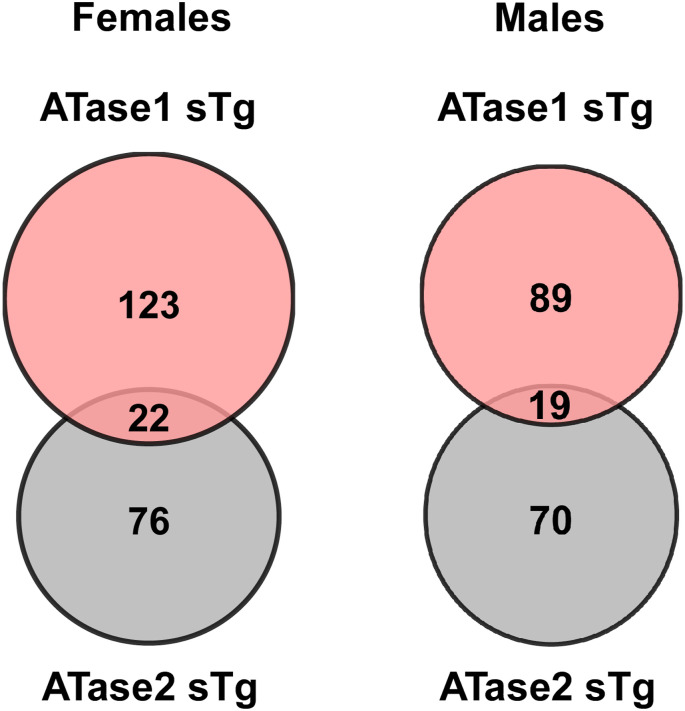
Venn diagrams of significantly changed glycoproteins in female and male ATase1 sTg and ATase2 sTg mouse cortex.

We found significant changes in 145 and 98 glycoforms of ATase1 sTg and ATase2 sTg females, and 108 and 89 glycoforms of the ATase1 sTg and ATase2 sTg males (Fig 7A). As with the proteome, the overlap among models were minimal, with 22 glycoproteins/glycoforms shared between ATase1 sTg and ATase2 sTg females and 19 between ATase1 sTg and ATase2 sTg males (Fig S4). Most of the observed changes were accounted for by sialic acid, fucose, and high mannose and were clustered within plasma membrane, cell surface, synapse, extracellular, and vesicle subgroups (Fig 7B). In essence, Golgi-dependent events and cellular compartments that depend on successful transition across the Golgi apparatus and toward the plasma membrane were affected.

**Figure S5.**
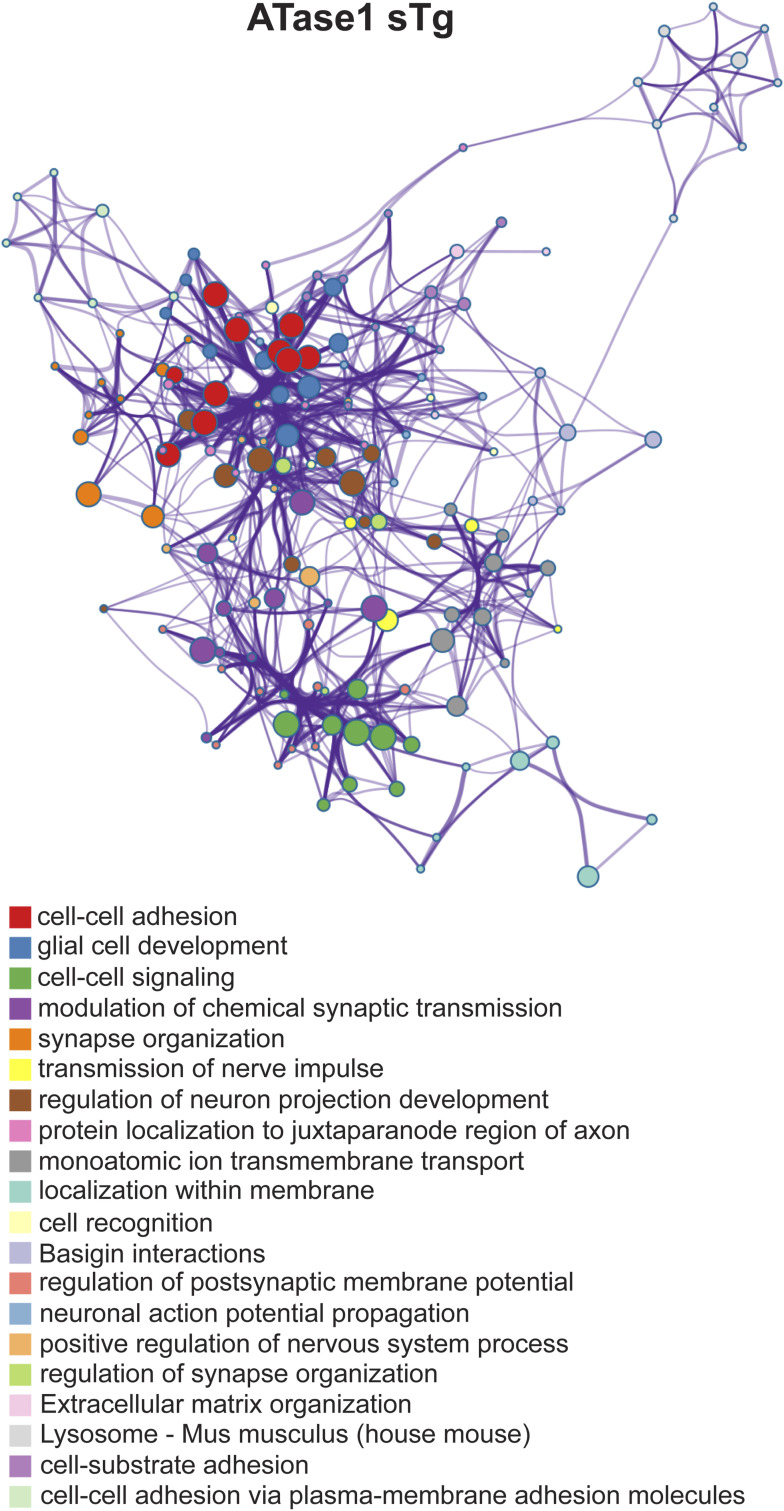
Enriched ontology cluster of significantly changed glycoproteins in ATase1 sTg mice. Clusters are identified by the different colors.

**Figure S6.**
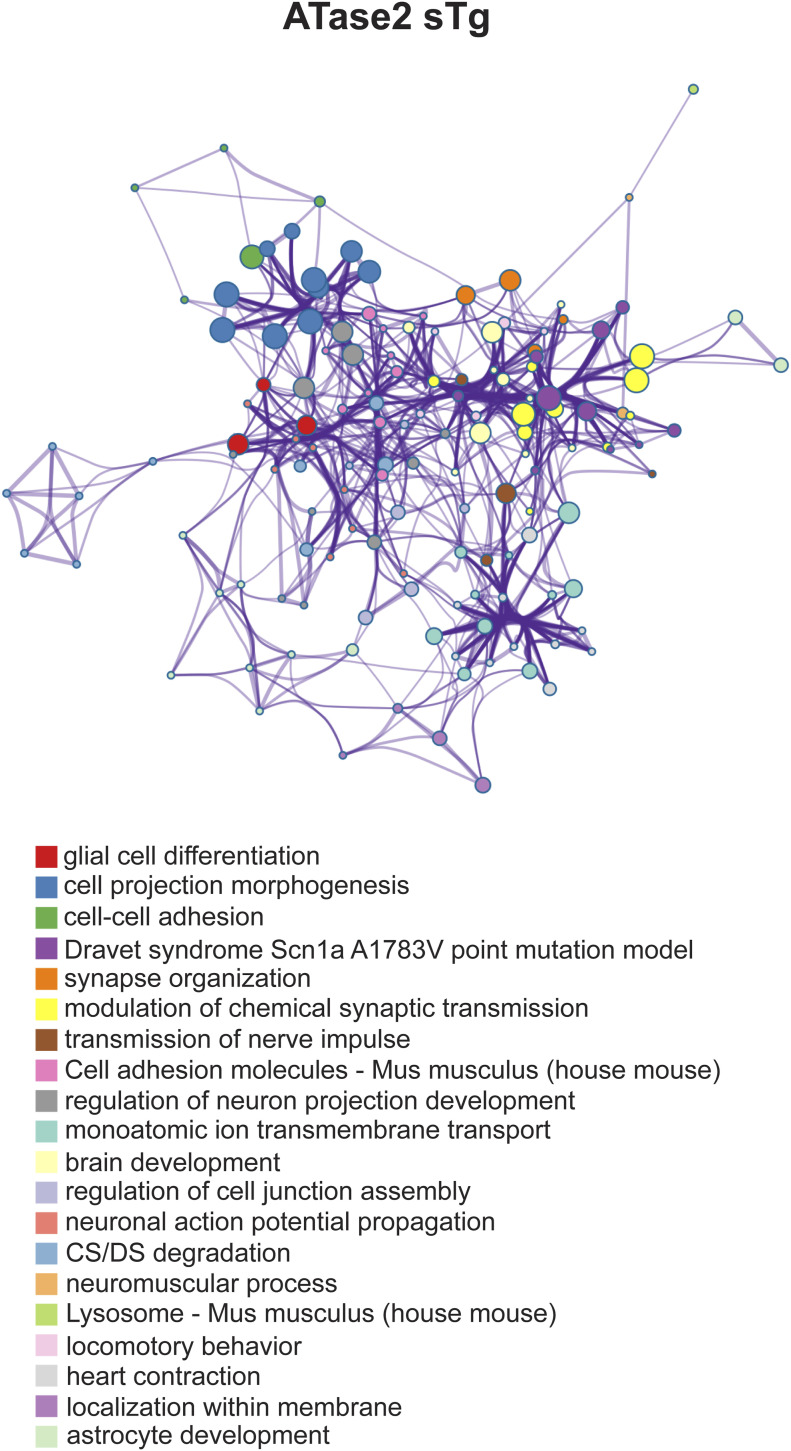
Enriched ontology cluster of significantly changed glycoproteins in ATase2 sTg mice. Clusters are identified by the different colors.

When we quantified all significant changes per glycosite, we found that most of the glycosites had one glycan structure affected with significant heterogeneity observed across models. Indeed ∼20% of glycosites had more than one glycan structure being significantly affected (Fig 7C). Furthermore, when we examined the glycan-type distribution as they pertain to the number of sites affected per protein, we observed a higher appearance of fucose on multiple sites per protein with an almost even distribution of sialic acid (Fig 7D). Collectively, these findings highlight the complexity of the N-glycan modifications caused by the overexpression of the ATases and point to specific changes of individual glycosylation events occurring at the ER-to-Golgi transition as well as within the Golgi apparatus. Finally, analysis of all significantly affected glycoproteins across the brain (cortex and hippocampus) points to fundamental biological functions that are intimately connected to neuronal biology and are highly dependent on dynamics of the secretory pathway (Fig S5 and S6). They included cell surface-dependent events, cell-cell adhesion/interactions, extracellular matrix organization, assembly and activity of synaptic terminals. These findings clearly implicate the ATases with the engagement and efficiency of the secretory pathway (discussed later).

### ATase1 sTg and ATase2 sTg mice display different engagement of selected pathways

**Figure 8.**
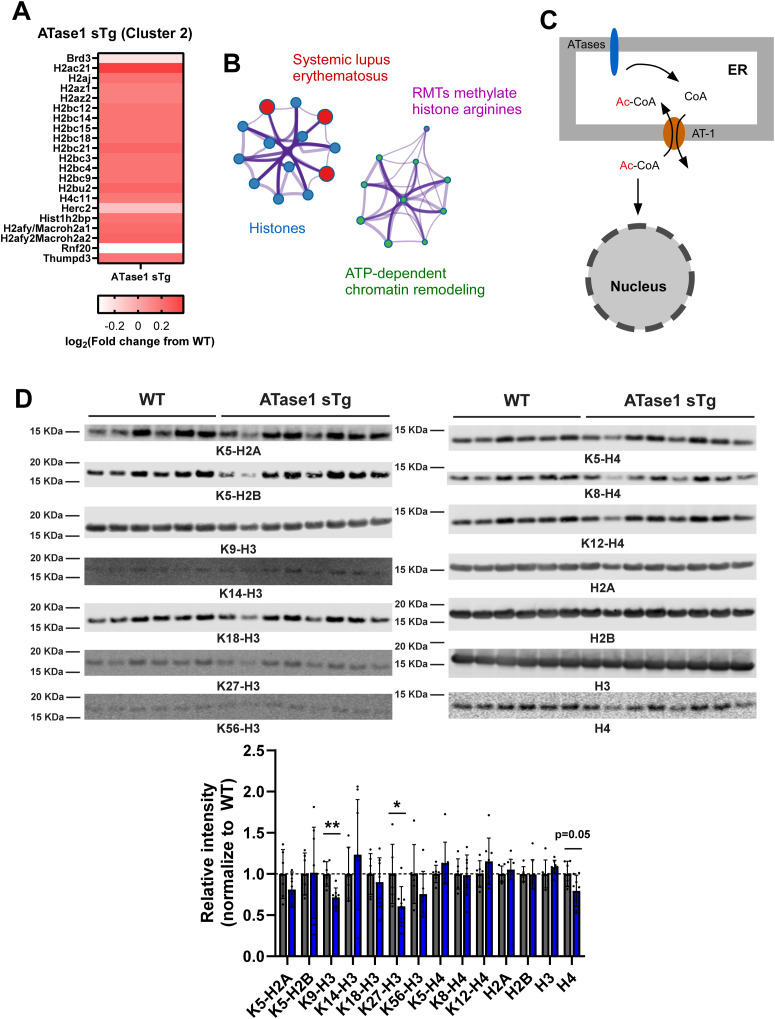
Nuclear adaptive response in ATase1 sTg mice. **(A, B)** Heatmap (A) and ontology enrichment (B) of cluster 2 from ATase1 sTg mice. Cluster labeling is shown in Fig 5C. **(C)** Changes in ATase activity in the ER lumen can affect the availability of acetyl-CoA in the cytosol and nucleus by regulating the antiporter activity of AT-1 (see also Fig 10). **(D)** Western blot and associated quantitation showing the histone acetylation profile of ATase1 sTg mice (brain cortex nuclear fraction; age: 6 mo). The number of animals is shown. *P < 0.05; **P < 0.005. Unpaired *t* test. Mice were ∼6 mo old when studied. Source data are available for this figure.

Next, we examined the engagement of selective pathways that appear to differentiate the ATase1 and ATase2 sTg models at the proteomic level. First, we targeted cluster 2 (nucleosome core/histones) in ATase1 sTg, which did not emerge from the Markov clustering of the ATase2 sTg dataset (Fig 5C). Most of ATase1 sTg cluster 2 proteins appeared to be up-regulated, when compared with WT mice (Fig 8A) and were further clustered under four different subcategories with histones and ATP-dependent chromatin remodeling proteins being the most represented (Fig 8B). Only three proteins appeared to be down-regulated (Fig 8A): Brd3, a bromodomain containing protein that recognizes and binds acetylated histones (LeRoy et al, 2008; Daneshvar et al, 2020); Herc2 and Rnf20, which might be involved in the regulation of chromosomal structure (Zhu et al, 2005).

As mentioned above, AT-1 acts as an antiporter by coupling the cytosol-to-ER flux of acetyl-CoA with the ER-to-cytosol flux of free CoA (Fig 8C). The antiporter activity is regulated by the availability of free CoA within the ER lumen, which depends on the acetyl-CoA:lysine acetyltransferase activity of the ATases (Fernandez-Fuente et al, 2023b). Therefore, it is possible that, in addition to changes in protein levels of nuclear/chromatin associated proteins, as reflected in Fig 8A, ATase1 sTg mice might display changes in the acetylation profile of core histone proteins caused by changes in acetyl-CoA availability in the cytosol and nucleus (Fig 8C). Immunoblot assessment revealed reduced acetylation of different histone marks (specifically: K5-H2A, K9-H3, K27-H3, and K56-H3), although only K9-H3 and K27-H3 reached statistical level of significance (Fig 8D). When taken together, the above results point to a marked adaptive nuclear response in ATase1 sTg mice, which was achieved through changes in protein levels and posttranslational modification of core histone proteins although the former mechanism appeared to be the most dominant.

**Figure 9.**
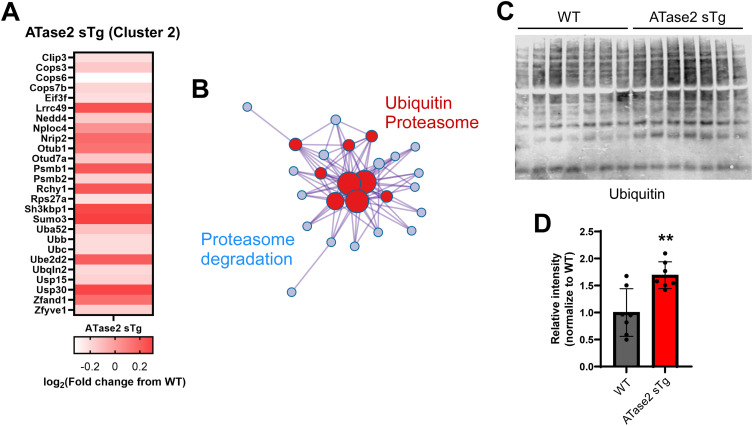
Adaptive response within the ubiquitin pathway in ATase2 sTg mice. **(A, B)** Heatmap (A) and ontology enrichment (B) of cluster 2 from ATase2 sTg mice. Cluster labeling is shown in Fig 5C. **(C, D)** Western blot (C) and associated quantitation (D) showing increased ubiquitination in ATase2 sTg (brain cortex; age: 3 mo). The number of animals is shown. **P < 0.005. Unpaired *t* test. Mice were ∼3 mo old when studied. Source data are available for this figure.

Next, we targeted cluster 2 (ubiquitin/proteasome/ER stress) in ATase2 sTg mice, which did not emerge from the Markov clustering of the ATase1 sTg dataset (Fig 5C). The two major subgroups within the cluster were related to the ubiquitin and proteasome machinery (Fig 9A and B). Indeed, there was a high representation of COP9 signalosome complex subunits, proteasome structural subunits, and different components of the ubiquitin system (Fig 9A and B). One of the biological functions currently associated with the ATase is the induction of autophagy from the ER (Fernandez-Fuente et al, 2023b). This was clearly documented in Atase1^−/−^ and Atase2^−/−^ mice, both displaying increased activation of the autophagy machinery (Rigby et al, 2021). Since a block in macroautophagy can result in increased activity of the ubiquitin/proteasome system (Komatsu et al, 2005, 2006; Behrends et al, 2010), we tested whether ATase2 sTg mice displayed increased levels of ubiquitin conjugated species. This was indeed the case, as documented by a generalized increase in the levels of ubiquitinated structures (Fig 9C and D).

In conclusion, systemic overexpression of either ATase1 or ATase2 in the mouse causes a similar but not identical progeria-like phenotype. The widespread proteomic alterations observed implicate the ATases with engagement of the secretory pathway, protein homeostasis, and metabolic crosstalk between different cellular compartments.

## Discussion

**Figure 10.**
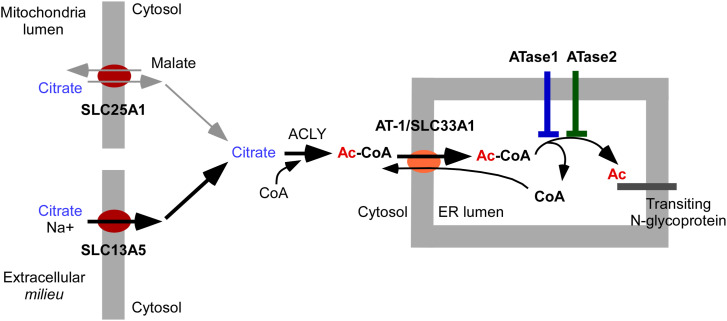
Schematic view of the citrate/acetyl-CoA pathway with the ER acetylation machinery. Description with relevant references is in the text. The ER acetylation machinery responds more dramatically to the citrate imported by SLC13A5 (black arrows) than SLC25A1 (gray arrows).

ATase1 and ATase2 are two ER-based acetyltransferases responsible for Nε-lysine acetylation of ER-cargo and -resident proteins. They work in concert with AT-1, an ER-membrane transporter, which ensures availability of acetyl-CoA into the ER lumen (Fig 10). Cytosolic acetyl-CoA mainly originates from citrate, through the ATP citrate lyase (ACLY), or acetate, through the acetyl-CoA synthetase 2 (ACSS2). The supply of citrate depends on SLC25A1, which exchanges citrate for malate across the mitochondrial membrane, and SLC13A5, which imports citrate and Na^+^ from the extracellular *milieu* (Fernandez-Fuente et al, 2023b). In the mouse, the ATases respond more dramatically to the citrate/acetyl-CoA flux, as dictated by the SLC13A5/AT-1 network (see Fig 10) (Fernandez-Fuente et al, 2023a). Indeed, systemic overexpression of SLC13A5 leads to up-regulation of endogenous AT-1, increased transport of acetyl-CoA into the ER lumen, and increased ATase-mediated acetylation of ER cargo and transiting proteins (Fernandez-Fuente et al, 2023a). The animals develop a multisystemic phenotype resembling segmental forms of human progerias. Both the hyperacetylation of ER cargo/transiting proteins and the progeria-like phenotype are rescued by the inhibition of the ATases (Fernandez-Fuente et al, 2023a). Neither the up-regulation of AT-1 nor the progeria-like phenotype is observed in mice with systemic overexpression of SLC25A1 (Fernandez-Fuente et al, 2023a). The differential behavior of SLC13A5 and SLC25A1 sTg mice is likely caused by their different transport properties, with the antiporter mechanism of SLC25A1 being limited by the availability of cytosolic malate (Fernandez-Fuente et al, 2023a). Systemic overexpression of AT-1 also leads to hyperacetylation of ER cargo/transiting proteins and a progeria-like phenotype that is rescued by the inhibition of the ATases (Peng et al, 2018; Murie et al, 2022). Finally, mice with systemic overexpression of ATase1 or ATase2 develop a multisystemic phenotype resembling segmental forms of human progerias (present study). Salient features included skin alterations, lordokyphosis, reduced bone density, splenomegaly, adenomegaly, and systemic inflammation (see Table 1). Overall, the phenotype of ATase1 and ATase2 sTg mice resemble the SLC13A5 sTg and AT-1 sTg phenotype (present study and [Peng et al, 2018; Fernandez-Fuente et al, 2023a]). In essence, the ATases appear to be the last output of the SLC13A5/AT-1 metabolic network that is intimately linked to a segmental form of progeria (see Fig 10). Support to this conclusion comes from human-based studies. Indeed, gene duplication events involving 17p13.1 (harboring SLC13A5), 3q25.31 (harboring AT-1), and 2p13.1 (harboring both ATases) are all associated with autism spectrum disorder with intellectual disability and progeria-like dysmorphism (see National Organization for Rare Disorders database; see also [Francke, 1978; Fineman et al, 1983; Fryns et al, 1989; Sawyer et al, 1994; Rizzu et al, 1997; Ounap et al, 2005; Krumm et al, 2013; Poultney et al, 2013; Carvalho et al, 2014; Mooneyham et al, 2014; Krumm et al, 2015]).

Progeroid syndromes are rare genetic disorders that manifest with delayed growth, facial dysmorphism and a complex multisystemic phenotype that mimics some features of an accelerated/pathogenic form of aging. Although with obvious limitations, they provide valuable models for studying the mechanisms of aging and age-associated diseases. Most studied forms of progerias are characterized by defects of the nuclear envelope or DNA instability. They include Werner syndrome, Hutchinson-Gilford syndrome, Cockaye syndrome, Xeroderma pigmentosum, Rothmund-Thomson syndrome, and Bloom syndrome. Both the genome instability and progeroid-like features are efficiently reproduced in the mouse (Bol et al, 1998; Chester et al, 1998; Lebel & Leder, 1998; Pendas et al, 2002; Osorio et al, 2011; Yokoyama et al, 2019). The mechanism underlying the SLC13A5, AT-1, and ATase duplication-associated syndromes differs substantially from the above and offers a unique model where specific metabolic outputs disrupt the proteostatic functions of the ER altering the quality of the secretome (discussed later). The position of the ATases as the last output of the pathway (Fig 10) might also offer mechanistic and therapeutic avenues for several age-associated diseases.

The entire ER acetylation machinery (AT-1 and the two ATases) has emerged as a novel branch of the more general nutrient-signaling pathway that ensures intracellular metabolic connectivity (Fernandez-Fuente et al, 2023b). Specifically, the ER acetylation machinery responds to the availability of acetyl-CoA to influence functional dynamics of the secretory pathway by regulating proteostasis within the ER/secretory pathway (Fernandez-Fuente et al, 2023b). This is clearly reflected by both the proteome and glycoproteome alterations observed in the brain of ATase1 and ATase2 sTg mice. At the level of the proteome, we found the largest changes within clusters that are broadly involved with the biosynthesis, folding, N-glycosylation, and quality control of nascent glycoproteins within the ER lumen (see Fig 5). At the level of the glycoproteome, we found significant alterations within post-ER events. This was reflected by changes in the levels of high mannose, fucosylated, and sialylated structures, which clearly point to altered ER-to-Golgi and intra-Golgi transition (see Figs 6D and 7). In essence, the proteome and glycoproteome datasets point to a converging outcome: overexpression of the ATases disrupts the ability of the ER to control the engagement of the secretory pathway and the quality of the *secretome*. On this regard, it is important to stress two concepts: (1) the quality of the *secretome*, which is embedded in the primary sequence of secreted proteins, depends on the ability of the cell to ensure required post-translational modifications and transport of nascent proteins to the right place; (2) N-glycosylation heavily influences protein activity, and defective N-glycosylation is the underlying basis of several congenital diseases (Sturla et al, 2001; Klaric & Lauc, 2022; Pasala et al, 2024; Raynor et al, 2024; Pan & Zhang, 2025). Consistently, the glycoproteome analysis of the ATase sTg models highlighted fundamental biological functions that are intimately connected to neuronal biology and are highly dependent on dynamics of the secretory pathway. They included cell surface-dependent events, cell-cell adhesion/interactions, extracellular matrix organization, assembly and activity of synaptic terminals (see Fig 7). These findings implicate the ATases with the engagement and efficiency of the secretory pathway.

The most notable differences within the proteome of ATase1 and ATase2 sTg mice were found with the nucleosome core/histone and ubiquitin/proteasome/quality control clusters with the former being represented in ATase1 sTg mice and the latter being represented in ATase2 sTg mice (see Fig 5C). Again, these differences are likely to reflect distinct biological attributes. Interestingly, both Atase1^−/−^ and Atase2^−/−^ mice displayed increased engagement of the autophagy machinery (Rigby et al, 2021). However, only Atase2^−/−^ mice displayed activation of the Atf6 canonical ER stress signaling pathway together with a more evident stimulation of macroautophagy (Rigby et al, 2021). Furthermore, the analysis of the acetyl-proteome of the knock-out models revealed a heavy predominance of proteasome categories in the Atase2^−/−^ model that did not appear in Atase1^−/−^ mice (Rigby et al, 2021). In essence, the knock-out and overexpressing models offer convergent insights into the differential biological functions of the ATases within the ER.

The acetyl-CoA:lysine acetyltransferase activity of the ATases yields free CoA, which can exit the ER through AT-1 (Fig 10) (Fernandez-Fuente et al, 2023b). The antiporter activity of AT-1 is regulated by the concentration gradients of both acetyl-CoA (in the cytosol) and free CoA (in the ER lumen). As a result, the activity of the two ATases can affect the antiporter activity of AT-1 by determining the levels of free CoA within the ER lumen (Rigby et al, 2021; Fernandez-Fuente et al, 2023b). While analyzing Atase1^−/−^ and Atase2^−/−^ mice, we noticed that the excess cytosolic acetyl-CoA accumulating from slowdown of the At-1 antiporter activity was being used differently in the two models: increased acetylation of non-ER/secretory proteins in Atase1^−/−^ mice and accumulation of lipid droplets in Atase2^−/−^ mice. A somewhat similar outcome emerged with the ATase1 sTg mice, which displayed a clear up-regulation of several histone and core nucleosome proteins together with reduced histone acetylation.

Overall, our results link a hyperactive ER acetylation machinery to a segmental form of progeria and reinforce the link between ER acetylation and proteostasis within the secretory pathway. Our study also reinforces the concept of biological divergent roles for ATase1 and ATase2. Finally, ATase1 sTg and ATase2 sTg mice, together with AT-1 sTg mice, offer unique insights on the molecular aspects of progeria and pathogenic forms of aging that differ from nuclear/genomic instability models.

## Materials and Methods

### Transgenic mouse generation

pTRE-ATase1 transgenic and pTRE-ATase2 transgenic mice were generated as described (Hullinger et al, 2016; Rigby et al, 2022a, 2022b). In brief, human cDNA was isolated from ATase1- and ATase2-pCMV6 plasmids (RC215647 for ATase1/NAT8B and RC202157 for ATase2/NAT8; Origene) using PCR and subsequently subcloned into pTRE-Tight plasmid (Takara Bio, Inc.). The resulting pTRE-Tight-ATase1 and pTRE-Tight-ATase2 plasmids were linearized with XhoI and then injected (3 ng/μl) into the pronuclei on one-cell C57BL/6J embryos (Stock No. 000664; The Jackson Laboratory). Rosa26:tTA; pTRE-ATase1 (ATase1 sTg) and Rosa26:tTA; pTRE-ATase2 (ATase2 sTg) were generated by crossing Rosa26:tTA mice with pTRE-ATase1 and pTRE-ATase2 mice, respectively. Genotyping from tail DNA was performed by TransnetYX using the following primers: Rosa26:tTA forward (5′-GCCGTGGGCCACTTCA-3′), Rosa26:tTA reverse (5′-CTGGTGCTCCTGGTCCTC-3′), pTRE-ATase1 forward (5′- GCTCGTTTAGTGAACCGTCAGAT-3′), pTRE-ATase1 reverse (5′- CTCCTGGTATTTGCGGATGTGAT-3′), pTRE-ATase2 forward (5′- GCTCGTTTAGTGAACCGTCAGAT-3′), and pTRE-ATase2 reverse (5′- CTCCTGGTATTTGCGGATGTGA-3′).

### Animals

All experimental animals were housed in the standardized cages provided by the University Laboratory Animal Resources and grouped with 1–5 mice per cage. Mice were supplied with either standard diets or diets with doxycycline (200 mg/kg) purchased from Bio-Serv and water ad libitum. All animal experiments were performed in accordance with the National Institutes of Health Guide for the Care and Use of Laboratory Animals and were approved by the Institutional Animal Care and Use Committee of the University of Wisconsin-Madison (protocol #M005120). Non-transgenic, WT C57BL/6J mice (Stock No. 000664; The Jackson Laboratory) were used as controls (WT). Age, sex, and number of animals used with each experiment are reported in the figure legends.

### Western blotting

Western blotting was conducted as previously described (Peng et al, 2016, 2018). The following primary antibodies were used in this study: β-actin (#3700, 1:5,000; Cell Signaling; #4967, 1:5,000; Cell Signaling), ATase1/NAT8B (#PA5-77166, 1:1,000; Invitrogen), ATase2/NAT8 (#NBP1-47863, 1:1,000; NOVUS), Acetyl-Histone H2A Lys5 (#2576, 1:1,000; Cell Signaling), Acetyl-Histone H2B Lys5 (#12799, 1:1,000; Cell Signaling), Acetyl-Histone H3 Lys9 (#9649, 1:1,000; Cell Signaling), Lys14 (#7627, 1:1,000; Cell Signaling), Lys18 (#13998, 1:1,000; Cell Signaling), Lys27 (#8173, 1:1,000; Cell Signaling), Lys56 (#4243, 1:1,000; Cell Signaling), Acetyl-Histone H4 Lys5 (#8647, 1:1,000; Cell Signaling), Lys8 (#2594, 1:1,000; Cell Signaling), Lys12 (#13944, 1:1,000; Cell Signaling), Histone H2A (#12349, 1:1,000; Cell Signaling), Histone H2B (#12364, 1:1,000; Cell Signaling), Histone H3 (#4499, 1:1,000; Cell Signaling), Histone H4 (#13919, 1:1,000; Cell Signaling), and Ubiquitin (#3936, 1:1,000; Cell Signaling). Both donkey anti-rabbit and goat anti mouse IRDye 800CW and 680RD-conjugated secondary antibodies (#926-32213; #926-68070; #926-32210; #926-68073; LI-COR) were used for infrared imaging (LI-COR Odyssey Infrared Imaging System; LI-COR Biosciences). For immunoglobin measurement, extracellular and intracellular fraction were prepared as previously described (Peng et al, 2018; Fernandez-Fuente et al, 2023a). In brief, liver tissue was homogenized in extracellular buffer (50 mM Tris–HCl, pH 7.6; 150 mM NaCl; 2 mM EDTA; 0.01 [wt/vol] SDS; and 0.01% [vol/vol] NP-40) with inhibitors. The supernatant was collected as extracellular fraction after centrifugation for 90 min at 17,142*g* with Eppendorf rotor FA-45-24-11-HS at 4°C. Pellet was then homogenized in intracellular buffer (50 mM Tris–HCl, pH 7.6; 150 mM NaCl; 2 mM EDTA and 0.01 [wt/vol] Triton X-100) with inhibitors. The supernatant was collected as intracellular fraction after centrifugation for 90 min at 17,142*g* with Eppendorf rotor FA-45-24-11-HS at 4°C. For nuclear fraction, target signals were normalized to total protein using Revert Total Protein Stain (#926-11021; LI-COR). Uncropped Western blot images are shown in the Supporting Data section.

### Bone density

Femur and Tibia were collected and fixed in 70% ethanol. Bone density was measured by the UltraFoxus DXA system (Faxitron) using the standard manufacturer protocols.

### Reverse transcription quantitative PCR (RT-qPCR)

Liver tissue was used for RNA extraction (#R1054; Zymo), cDNA synthesis (#18080400; Invirogen) and quantitative PCR using Roche 480 LightCycler and SYBR Green I Master (#04887352001; Roche). Expression levels were normalized to β-actin. Relative expression levels were normalized to control and expressed as fold change. The following primers were used: β-actin forward (5′-CTA​AGG​CCA​ACC​GTG​AAA​AG-3′), β-actin reverse (5′-ACC​AGA​GGC​ATA​CAG​GGA​CA-3′), p16 forward (5′-GTG​TGC​ATG​ACG​TGC​GGG-3′), p16 reverse (5′-GCA​GTT​CGA​ATC​TGC​ACC​GTA​G-3′), p21 forward (5′-AAC​ATC​TCA​GGG​CCG​AAA-3′), p21 reverse (5′-TGC​GCT​TGG​AGT​GAT​AGA​AA-3′).

### Senescence β-galactosidase assay

Senescence β-Galactosidase was detected by using Senescence β-galactosidase Staining Kit (#9860S; Cell signaling Technology). In brief, mouse liver cryosections (10 μm) were fixed and stained with β-galactosidase at 37°C overnight.

### Histology

Samples for H&E staining were immediately fixed with 10% neutral buffered formalin overnight after collection. Samples were then embedded in paraffin by standard techniques and section on microtome. After deparaffinization and rehydration, sections were stained with H&E staining.

### 12-plex DiLeu labeling

12-plex dimethylated leucine (DiLeu) labeling was performed as previously described (Frost et al, 2015; Dieterich et al, 2021). Briefly, dissected brain samples of ATase1 sTg, ATase2 sTg, and WT littermates were homogenized and lysed in 8 M urea buffer with a probe sonicator. The extracted proteins were reduced by 5 mM DTT at RT for 1 h, followed by alkylation with 15 mM IAA for 30 min in the dark. The alkylation reaction was quenched by adding DTT to a final concentration of 5 mM. The alkylated proteins were then diluted and digested with trypsin at an enzyme-to-protein ratio of 1:50 at 37°C for 18 h. The resulting tryptic peptides were desalted using C18 SepPak cartridges, dried under vacuum, and reconstituted in 0.5 M TEAB for labeling. DiLeu tags were dissolved in anhydrous DMF and activated with DMTMM and NMM at a 0.6× molar ratio relative to the tags. The mixture was vortexed at RT for 1 h. The reaction mixture was vortexed at RT for 1 h. After centrifugation, the supernatant was immediately mixed with tryptic peptides from a single condition at a 10:1 tag-to-peptide (wt/wt) ratio and incubated with vortexing at RT for 2 h. The reaction was quenched by NH_2_OH. Labeled peptide batches were combined to generate 12-plex mixtures. An aliquot of the dried peptides was further processed using SCX spin tips according to the manufacturer’s protocol.

### High pH fractionation

High-pH (HpH) fractionation was conducted using a Waters Alliance e2695 HPLC equipped with a C18 reversed-phase column (2.1 × 150 mm^2^, 5 μm, 100 Å) at a flow rate of 0.2 ml/min. Mobile phase A consisted of 10 mM ammonium formate at pH 10 adjusted with ammonium hydroxide while mobile phase B consisted of 90% ACN and 10 mM ammonium formate at pH 10. Separation was achieved using the following gradient: 1% B (0–5 min), 1–40% B (5–50 min), 40–60% B (50–54 min), 60–70% B (54–58 min), and 70–100% B (58–59 min). Fractions were collected every 2 min, and nonadjacent fractions were pooled into eight groups before being dried under vacuum for subsequent LC-MS/MS analysis.

### Glycopeptide enrichment

DiLeu labeled glycopeptides were enriched using in-house packed strong anion-exchange (SAX)-HILIC SPE tips following a previously reported protocol with minor modifications (Selman et al, 2011). 3 mg of cotton wool was inserted into an empty TopTip. PolySAX LP bulk material was prepared as a 10 mg/200 μl slurry in 1% TFA and activated by vigorous vortexing for 15 min. The activated slurry was then transferred to the spin tip at a beads-to-peptide mass ratio of 30:1. Solvent removal was performed by centrifugation at 121*g* with Eppendorf rotor F45-24-11 for 2 min, ensuring the SAX material was firmly packed at the top of the tip. The stationary phase was conditioned with 300 μl of 1% TFA and 300 μl of loading buffer (80% ACN, 1% TFA), with each step repeated three times. DiLeu-labeled peptides were aliquoted to a total of 1,800 μg, with each aliquot dissolved in 300 μl of loading buffer before loading onto the tips by centrifugation at 121*g* with Eppendorf rotor F45-24-11 for 2 min. The flow-through was collected and reloaded to ensure complete peptide retention. The tips were then washed six times with 300 μl of loading buffer. Elution was performed sequentially in four fractions using 300 μl of 70% ACN with 0.1% FA, 50% ACN with 0.1% FA, 25% ACN with 0.1% FA, and 0% ACN with 0.1% FA. Each eluted fraction was collected separately and dried under vacuum before MS analysis.

### LC–MS/MS analysis

Enriched glycopeptides from each fraction were reconstituted in 0.1% FA and analyzed by reversed-phase LC-MS/MS with an Orbitrap Fusion Lumus coupled to a Dionex UltiMate 3000 UPLC system. Peptides separation was performed on an 18 cm × 75 μm i.d. custom-packed BEH C18 (1.7 μm, 130 Å) capillary column using an 80-min gradient from 0% to 30% ACN containing 0.1% FA. Data acquisition was carried out in top-speed mode with a 3-s cycle time. Precursor ion scans were recorded over an m/z range of 400–2,000 at a resolution of 60,000, with a normalized automatic gain control (AGC) target of 60% and a maximum injection time (IT) of 200 ms. Selected precursor ions underwent higher-energy C-trap dissociation (HCD) with a normalized collision energy (NCE) of 30, incorporating a ±3% stepped HCD collision energy. Tandem MS spectra were acquired at a resolution of 60,000 with a lower mass limit of m/z 110. A dynamic exclusion of 12 s with a 10 ppm mass tolerance was applied.

Peptides from each fraction were reconstituted in 0.1% FA and analyzed using a Dionex UltiMate 3000 UPLC coupled to a Q Exactive HF Orbitrap mass spectrometer. Peptides separation was performed using the same column as described above, with a 100-min gradient from 0 to 30% ACN containing 0.1% FA. The MS scan range covered *m/z* 300–1,500, also at a resolution of 60,000, with an AGC target of 1E6 and a maximum IT of 100 ms. The MS/MS method used a top 20 data-dependent acquisition (DDA) mode, with all MS/MS dissociations conducted using an NCE of 30. The MS/MS parameters included a resolution of 60,000, an AGC target of 1E5, and a maximum IT of 200 ms. A dynamic exclusion of 45 s with an isolation window of 1.0 *m/z* was applied.

### Glycoproteomics and proteomics data analysis

Raw files were processed using the Byonic search engine integrated within Proteome Discoverer 2.5. Spectra were searched against the SwissProt *Mus musculus* proteome database (12 January 2021). Trypsin digestion was allowed with up to two missed cleavages. The parent mass tolerance was set to 10 ppm, whereas the fragment mass tolerance was 0.01 D. Fixed modifications included as carbamidomethylation (+57.02146 D) on C residues and 12-plex DiLeu (+145.12801 D) on peptide N-terminus and K. For N-glycoproteomics, dynamic modifications included oxidation of M (+15.99492 D), deamidation (+0.984016 D) of N or Q, and N-glycosylation. Glycan modifications were specified as Byonic embedded mammalian N-glycan database (309 entries). Protein identifications were filtered to a 1% false discovery rate (FDR). Glycopeptides were categorized into five glycan types based on their composition: (1) sialic acid (containing NeuAc/NeuGc), (2) fucose (containing Fucose), (3) complex/hybrid (>2 NeuAc), (4) high-mannose (2 NeuAc and >5 Hex), and (5) paucimannose (2 NeuAc and <5 Hex). Peptide identification results were filtered at Byonic score >100, PEP 2D < 0.05, and |Log Prob| > 1. For proteomics, fixed and dynamic modifications are the same as N-glycoproteomics, the same fixed and dynamic modifications as in N-glycoproteomics were applied, except for the exclusion of N-glycosylation as a dynamic modification. Only high-confidence protein identifications were used for further analysis. Reporter ion intensities from the 12-plex DiLeu channels were median-normalized in Perseus to correct for systematic biases (Tyanova et al, 2016). Gene ontology annotation and *t* test of quantitation results were performed using Perseus (Tyanova et al, 2016). Further data processing was conducted using custom Python and R scripts. Outputs were analyzed with Metascape (Zhou et al, 2019). The LC-MS/MS raw data and annotated spectra have been deposited to the Proteome Xchange Consortium via the MassIVE partner repository with the accession number “MSV000097367”.

### Nuclear enrichment

Nuclear fractions were prepared by using EpiQuik Nuclear Extraction Kit (#OP-0002-1; EpiGentek). Briefly, Mouse cortex was homogenized in NE1 buffer containing 0.1% DTT and centrifuged for 10 min at 14,167*g* with Eppendorf rotor FA-45-24-11-HS at 4°C. The pellet was homogenized in NE2 buffer containing 0.1% DTT and PIC by vortex and sonication, and further centrifuged for 10 min at 19,283*g* with Eppendorf rotor FA-45-24-11-HS at 4°C. Finally, the supernatant was collected as nuclear fraction for further experiments.

### Statistics

Specific sample size was described in each figure legend. GraphPad Prism 10.0.2 was used to analyze all experimental data. Data were presented as mean ± SD with all the individual data points. Unpaired *t* test or Welch’*t* test were used to compare the statistical difference between two groups. Statistical significance was presented as following: *P < 0.05, **P < 0.005, ***P < 0.0005, ****P < 0.0001.

## Supplementary Material

Reviewer comments

## Data Availability

The LC-MS/MS raw data and annotated spectra have been deposited to the ProteomeXchange Consortium via the MassIVE partner repository with the accession number “MSV000097367.” Source data for the graphs and charts are available as an excel file labeled as “Data points” and any remaining information can be obtained from the corresponding author upon reasonable request.

## References

[bib1] Behrends C, Sowa ME, Gygi SP, Harper JW (2010) Network organization of the human autophagy system. Nature 466: 68–76. 10.1038/nature09204 20562859 PMC2901998

[bib2] Bol SA, van Steeg H, Jansen JG, Van Oostrom C, de Vries A, de Groot AJ, Tates AD, Vrieling H, van Zeeland AA, Mullenders LH (1998) Elevated frequencies of benzo(a)pyrene-induced Hprt mutations in internal tissue of XPA-deficient mice. Cancer Res 58: 2850–2856. 9661901

[bib3] Carvalho CM, Vasanth S, Shinawi M, Russell C, Ramocki MB, Brown CW, Graakjaer J, Skytte AB, Vianna-Morgante AM, Krepischi AC, (2014) Dosage changes of a segment at 17p13.1 lead to intellectual disability and microcephaly as a result of complex genetic interaction of multiple genes. Am J Hum Genet 95: 565–578. 10.1016/j.ajhg.2014.10.006 25439725 PMC4225592

[bib4] Chester N, Kuo F, Kozak C, O’Hara CD, Leder P (1998) Stage-specific apoptosis, developmental delay, and embryonic lethality in mice homozygous for a targeted disruption in the murine Bloom’s syndrome gene. Genes Dev 12: 3382–3393. 10.1101/gad.12.21.3382 9808625 PMC317228

[bib5] Daneshvar K, Ardehali MB, Klein IA, Hsieh FK, Kratkiewicz AJ, Mahpour A, Cancelliere SOL, Zhou C, Cook BM, Li W, (2020) lncRNA DIGIT and BRD3 protein form phase-separated condensates to regulate endoderm differentiation. Nat Cell Biol 22: 1211–1222. 10.1038/s41556-020-0572-2 32895492 PMC8008247

[bib6] Dieterich IA, Cui Y, Braun MM, Lawton AJ, Robinson NH, Peotter JL, Yu Q, Casler JC, Glick BS, Audhya A, (2021) Acetyl-CoA flux from the cytosol to the ER regulates engagement and quality of the secretory pathway. Sci Rep 11: 2013. 10.1038/s41598-021-81447-6 33479349 PMC7820588

[bib7] Ding Y, Dellisanti CD, Ko MH, Czajkowski C, Puglielli L (2014) The endoplasmic reticulum-based acetyltransferases, ATase1 and ATase2, associate with the oligosaccharyltransferase to acetylate correctly folded polypeptides. J Biol Chem 289: 32044–32055. 10.1074/jbc.M114.585547 25301944 PMC4231681

[bib8] Farrugia MA, Puglielli L (2018) Nε-lysine acetylation in the endoplasmic reticulum - a novel cellular mechanism that regulates proteostasis and autophagy. J Cell Sci 131: jcs221747. 10.1242/jcs.221747 30446507 PMC6262770

[bib9] Fernandez-Fuente G, Overmyer KA, Lawton AJ, Kasza I, Shapiro SL, Gallego-Muñoz P, Coon JJ, Denu JM, Alexander CM, Puglielli L (2023a) The citrate transporters SLC13A5 and SLC25A1 elicit different metabolic responses and phenotypes in the mouse. Commun Biol 6: 926. 10.1038/s42003-023-05311-1 37689798 PMC10492862

[bib10] Fernandez-Fuente G, Rigby MJ, Puglielli L (2023b) Intracellular Citrate/acetyl-CoA flux and endoplasmic reticulum acetylation: Connectivity is the answer. Mol Metab 67: 101653. 10.1016/j.molmet.2022.101653 36513219 PMC9792894

[bib11] Fineman RM, Buyse M, Morgan M (1983) Variable phenotype associated with duplication of different regions of 2p. Am J Med Genet 15: 451–456. 10.1002/ajmg.1320150310 6881212

[bib12] Francke U (1978) Clinical syndromes associated with partial duplications of chromosomes 2 and 3: dup(2p),dup(2q),dup(3p),dup(3q). Birth Defects Orig Artic Ser 14: 191–217. 365267

[bib13] Frost DC, Greer T, Li L (2015) High-resolution enabled 12-plex DiLeu isobaric tags for quantitative proteomics. Anal Chem 87: 1646–1654. 10.1021/ac503276z 25405479 PMC4318621

[bib14] Fryns JP, Kleczkowska A, Kenis H, Decock P, Van den Berghe H (1989) Partial duplication of the short arm of chromosome 2 (dup(2)(p13--p21) associated with mental retardation and an Aarskog-like phenotype. Ann Genet 32: 174–176. 2573314

[bib15] Gonzalo S, Kreienkamp R, Askjaer P (2017) Hutchinson-gilford progeria syndrome: A premature aging disease caused by LMNA gene mutations. Ageing Res Rev 33: 18–29. 10.1016/j.arr.2016.06.007 27374873 PMC5195863

[bib16] Hirschberg CB, Robbins PW, Abeijon C (1998) Transporters of nucleotide sugars, ATP, and nucleotide sulfate in the endoplasmic reticulum and Golgi apparatus. Annu Rev Biochem 67: 49–69. 10.1146/annurev.biochem.67.1.49 9759482

[bib17] Hullinger R, Li M, Wang J, Peng Y, Dowell JA, Bomba-Warczak E, Mitchell HA, Burger C, Chapman ER, Denu JM, (2016) Increased expression of AT-1/SLC33A1 causes an autistic-like phenotype in mice by affecting dendritic branching and spine formation. J Exp Med 213: 1267–1284. 10.1084/jem.20151776 27242167 PMC4925020

[bib18] Karikkineth AC, Scheibye-Knudsen M, Fivenson E, Croteau DL, Bohr VA (2017) Cockayne syndrome: Clinical features, model systems and pathways. Ageing Res Rev 33: 3–17. 10.1016/j.arr.2016.08.002 27507608 PMC5195851

[bib19] Klarić TS, Lauc G (2022) The dynamic brain N-glycome. Glycoconj J 39: 443–471. 10.1007/s10719-022-10055-x 35334027

[bib20] Komatsu M, Waguri S, Ueno T, Iwata J, Murata S, Tanida I, Ezaki J, Mizushima N, Ohsumi Y, Uchiyama Y, (2005) Impairment of starvation-induced and constitutive autophagy in Atg7-deficient mice. J Cell Biol 169: 425–434. 10.1083/jcb.200412022 15866887 PMC2171928

[bib21] Komatsu M, Waguri S, Chiba T, Murata S, Iwata J, Tanida I, Ueno T, Koike M, Uchiyama Y, Kominami E, (2006) Loss of autophagy in the central nervous system causes neurodegeneration in mice. Nature 441: 880–884. 10.1038/nature04723 16625205

[bib22] Krumm N, O’Roak BJ, Karakoc E, Mohajeri K, Nelson B, Vives L, Jacquemont S, Munson J, Bernier R, Eichler EE (2013) Transmission disequilibrium of small CNVs in simplex autism. Am J Hum Genet 93: 595–606. 10.1016/j.ajhg.2013.07.024 24035194 PMC3791263

[bib23] Krumm N, Turner TN, Baker C, Vives L, Mohajeri K, Witherspoon K, Raja A, Coe BP, Stessman HA, He ZX, (2015) Excess of rare, inherited truncating mutations in autism. Nat Genet 47: 582–588. 10.1038/ng.3303 25961944 PMC4449286

[bib24] Lebel M, Leder P (1998) A deletion within the murine Werner syndrome helicase induces sensitivity to inhibitors of topoisomerase and loss of cellular proliferative capacity. Proc Natl Acad Sci U S A 95: 13097–13102. 10.1073/pnas.95.22.13097 9789047 PMC23722

[bib25] LeRoy G, Rickards B, Flint SJ (2008) The double bromodomain proteins Brd2 and Brd3 couple histone acetylation to transcription. Mol Cell 30: 51–60. 10.1016/j.molcel.2008.01.018 18406326 PMC2387119

[bib26] Liao CY, Kennedy BK (2014) Mouse models and aging: Longevity and progeria. Curr Top Dev Biol 109: 249–285. 10.1016/B978-0-12-397920-9.00003-2 24947239

[bib27] Liu P, Jiang B, Ma J, Lin P, Zhang Y, Shao C, Sun W, Gong Y (2017) S113R mutation in SLC33A1 leads to neurodegeneration and augmented BMP signaling in a mouse model. Dis Model Mech 10: 53–62. 10.1242/dmm.026880 27935820 PMC5278525

[bib28] Mooneyham KA, Holden KR, Cathey S, Dwivedi A, Dupont BR, Lyons MJ (2014) Neurodevelopmental delays and macrocephaly in 17p13.1 microduplication syndrome. Am J Med Genet A 164A: 2887–2891. 10.1002/ajmg.a.36708 25123844

[bib29] Murie M, Peng Y, Rigby MJ, Dieterich IA, Farrugia MA, Endresen A, Bhattacharyya A, Puglielli L (2022) ATase inhibition rescues age-associated proteotoxicity of the secretory pathway. Commun Biol 5: 173. 10.1038/s42003-022-03118-0 35217767 PMC8881600

[bib30] Osorio FG, Navarro CL, Cadiñanos J, López-Mejía IC, Quirós PM, Bartoli C, Rivera J, Tazi J, Guzmán G, Varela I, (2011) Splicing-directed therapy in a new mouse model of human accelerated aging. Sci Transl Med 3: 106ra107. 10.1126/scitranslmed.3002847 22030750

[bib31] Ounap K, Ilus T, Bartsch O (2005) A girl with inverted triplication of chromosome 3q25.3 -> q29 and multiple congenital anomalies consistent with 3q duplication syndrome. Am J Med Genet A 134: 434–438. 10.1002/ajmg.a.30134 15793836

[bib32] Pan Q, Zhang XL (2025) Roles of core fucosylation modification in immune system and diseases. Cell Insight 4: 100211. 10.1016/j.cellin.2024.100211 39624801 PMC11609374

[bib33] Pasala C, Sharma S, Roychowdhury T, Moroni E, Colombo G, Chiosis G (2024) N-glycosylation as a modulator of protein conformation and assembly in disease. Biomolecules 14: 282. 10.3390/biom14030282 38540703 PMC10968129

[bib34] Pehar M, Lehnus M, Karst A, Puglielli L (2012) Proteomic assessment shows that many endoplasmic reticulum (ER)-resident proteins are targeted by N(epsilon)-lysine acetylation in the lumen of the organelle and predicts broad biological impact. J Biol Chem 287: 22436–22440. 10.1074/jbc.C112.362871 22628546 PMC3391156

[bib35] Pendás AM, Zhou Z, Cadiñanos J, Freije JM, Wang J, Hultenby K, Astudillo A, Wernerson A, Rodríguez F, Tryggvason K, (2002) Defective prelamin A processing and muscular and adipocyte alterations in Zmpste24 metalloproteinase-deficient mice. Nat Genet 31: 94–99. 10.1038/ng871 11923874

[bib36] Peng Y, Li M, Clarkson BD, Pehar M, Lao PJ, Hillmer AT, Barnhart TE, Christian BT, Mitchell HA, Bendlin BB, (2014) Deficient import of acetyl-CoA into the ER lumen causes neurodegeneration and propensity to infections, inflammation, and cancer. J Neurosci 34: 6772–6789. 10.1523/JNEUROSCI.0077-14.2014 24828632 PMC4019794

[bib37] Peng Y, Kim MJ, Hullinger R, O’Riordan KJ, Burger C, Pehar M, Puglielli L (2016) Improved proteostasis in the secretory pathway rescues Alzheimer’s disease in the mouse. Brain 139: 937–952. 10.1093/brain/awv385 26787453 PMC4805081

[bib38] Peng Y, Shapiro SL, Banduseela VC, Dieterich IA, Hewitt KJ, Bresnick EH, Kong G, Zhang J, Schueler KL, Keller MP, (2018) Increased transport of acetyl-CoA into the endoplasmic reticulum causes a progeria-like phenotype. Aging Cell 17: e12820. 10.1111/acel.12820 30051577 PMC6156544

[bib39] Pivnick EK, Angle B, Kaufman RA, Hall BD, Pitukcheewanont P, Hersh JH, Fowlkes JL, Sanders LP, O’Brien JM, Carroll GS, (2000) Neonatal progeroid (Wiedemann-Rautenstrauch) syndrome: Report of five new cases and review. Am J Med Genet 90: 131–140. 10.1002/(sici)1096-8628(20000117)90:2<131::aid-ajmg9>3.0.co;2-e 10607952

[bib40] Poultney CS, Goldberg AP, Drapeau E, Kou Y, Harony-Nicolas H, Kajiwara Y, De Rubeis S, Durand S, Stevens C, Rehnström K, (2013) Identification of small exonic CNV from whole-exome sequence data and application to autism spectrum disorder. Am J Hum Genet 93: 607–619. 10.1016/j.ajhg.2013.09.001 24094742 PMC3791269

[bib41] Puglielli L, Rigby MJ, Fernandez-Fuente G (2023) Acetyl-CoA and acetylation in biology and disease. In Cellular Lipid in Health and Disease, Ntambi JM (ed), pp 483–506. Amsterdam, Netherlands: Elsevier Inc.

[bib42] Raynor A, Haouari W, Lebredonchel E, Foulquier F, Fenaille F, Bruneel A (2024) Biochemical diagnosis of congenital disorders of glycosylation. Adv Clin Chem 120: 1–43. 10.1016/bs.acc.2024.03.001 38762238

[bib43] Rigby MJ, Ding Y, Farrugia MA, Feig M, Cortese GP, Mitchell H, Burger C, Puglielli L (2020) The endoplasmic reticulum acetyltransferases ATase1/NAT8B and ATase2/NAT8 are differentially regulated to adjust engagement of the secretory pathway. J Neurochem 154: 404–423. 10.1111/jnc.14958 31945187 PMC7363514

[bib44] Rigby MJ, Lawton AJ, Kaur G, Banduseela VC, Kamm WE, Lakkaraju A, Denu JM, Puglielli L (2021) Endoplasmic reticulum acetyltransferases Atase1 and Atase2 differentially regulate reticulophagy, macroautophagy and cellular acetyl-CoA metabolism. Commun Biol 4: 454. 10.1038/s42003-021-01992-8 33846551 PMC8041774

[bib45] Rigby MJ, Orefice NS, Lawton AJ, Ma M, Shapiro SL, Yi SY, Dieterich IA, Frelka A, Miles HN, Pearce RA, (2022a) Increased expression of SLC25A1/CIC causes an autistic-like phenotype with altered neuron morphology. Brain 145: 500–516. 10.1093/brain/awab295 35203088 PMC9014753

[bib46] Rigby MJ, Orefice NS, Lawton AJ, Ma M, Shapiro SL, Yi SY, Dieterich IA, Frelka A, Miles HN, Pearce RA, (2022b) SLC13A5/sodium-citrate co-transporter overexpression causes disrupted white matter integrity and an autistic-like phenotype. Brain Commun 4: fcac002. 10.1093/braincomms/fcac002 35146426 PMC8823335

[bib47] Rizzu P, Haddad BR, Vallcorba I, Alonso A, Ferro MT, Garcia-Sagredo JM, Baldini A (1997) Delineation of a duplication map of chromosome 3q: A new case confirms the exclusion of 3q25-q26.2 from the duplication 3q syndrome critical region. Am J Med Genet 68: 428–432. 10.1002/(sici)1096-8628(19970211)68:4<428::aid-ajmg11>3.0.co;2-u 9021016

[bib48] Sawyer JR, Jones E, Hawks FF, Quirk JG, Jr., Cunniff C (1994) Duplication and deletion of chromosome band 2(p21p22) resulting from a familial interstitial insertion (2;11)(p21;p15). Am J Med Genet 49: 422–427. 10.1002/ajmg.1320490414 8160737

[bib49] Selman MH, Hemayatkar M, Deelder AM, Wuhrer M (2011) Cotton HILIC SPE microtips for microscale purification and enrichment of glycans and glycopeptides. Anal Chem 83: 2492–2499. 10.1021/ac1027116 21366235

[bib50] Sturla L, Puglielli L, Tonetti M, Berninsone P, Hirschberg CB, De Flora A, Etzioni A (2001) Impairment of the Golgi GDP-L-fucose transport and unresponsiveness to fucose replacement therapy in LAD II patients. Pediatr Res 49: 537–542. 10.1203/00006450-200104000-00016 11264438

[bib51] Tyanova S, Temu T, Sinitcyn P, Carlson A, Hein MY, Geiger T, Mann M, Cox J (2016) The perseus computational platform for comprehensive analysis of (prote)omics data. Nat Methods 13: 731–740. 10.1038/nmeth.3901 27348712

[bib52] Yokoyama H, Moreno-Andres D, Astrinidis SA, Hao Y, Weberruss M, Schellhaus AK, Lue H, Haramoto Y, Gruss OJ, Antonin W (2019) Chromosome alignment maintenance requires the MAP RECQL4, mutated in the Rothmund-Thomson syndrome. Life Sci Alliance 2: e201800120. 10.26508/lsa.201800120 30718377 PMC6362308

[bib53] Zhou Y, Zhou B, Pache L, Chang M, Khodabakhshi AH, Tanaseichuk O, Benner C, Chanda SK (2019) Metascape provides a biologist-oriented resource for the analysis of systems-level datasets. Nat Commun 10: 1523. 10.1038/s41467-019-09234-6 30944313 PMC6447622

[bib54] Zhu B, Zheng Y, Pham AD, Mandal SS, Erdjument-Bromage H, Tempst P, Reinberg D (2005) Monoubiquitination of human histone H2B: The factors involved and their roles in HOX gene regulation. Mol Cell 20: 601–611. 10.1016/j.molcel.2005.09.025 16307923

