# Supplementary material for: Overexpression of ATase1 and ATase2 disrupts the secretome and causes a progeria phenotype
Source: Life Sci Alliance. 2025 Sep 10;8(12):e202503378. doi: 10.26508/lsa.202503378 (PMC12423556; doi:10.26508/lsa.202503378)
Supplement: Supplementary file 1 [file LSA-2025-03378_SdataF1.pdf]

SOURCE DATA 1

Original Western blots from Figure 1

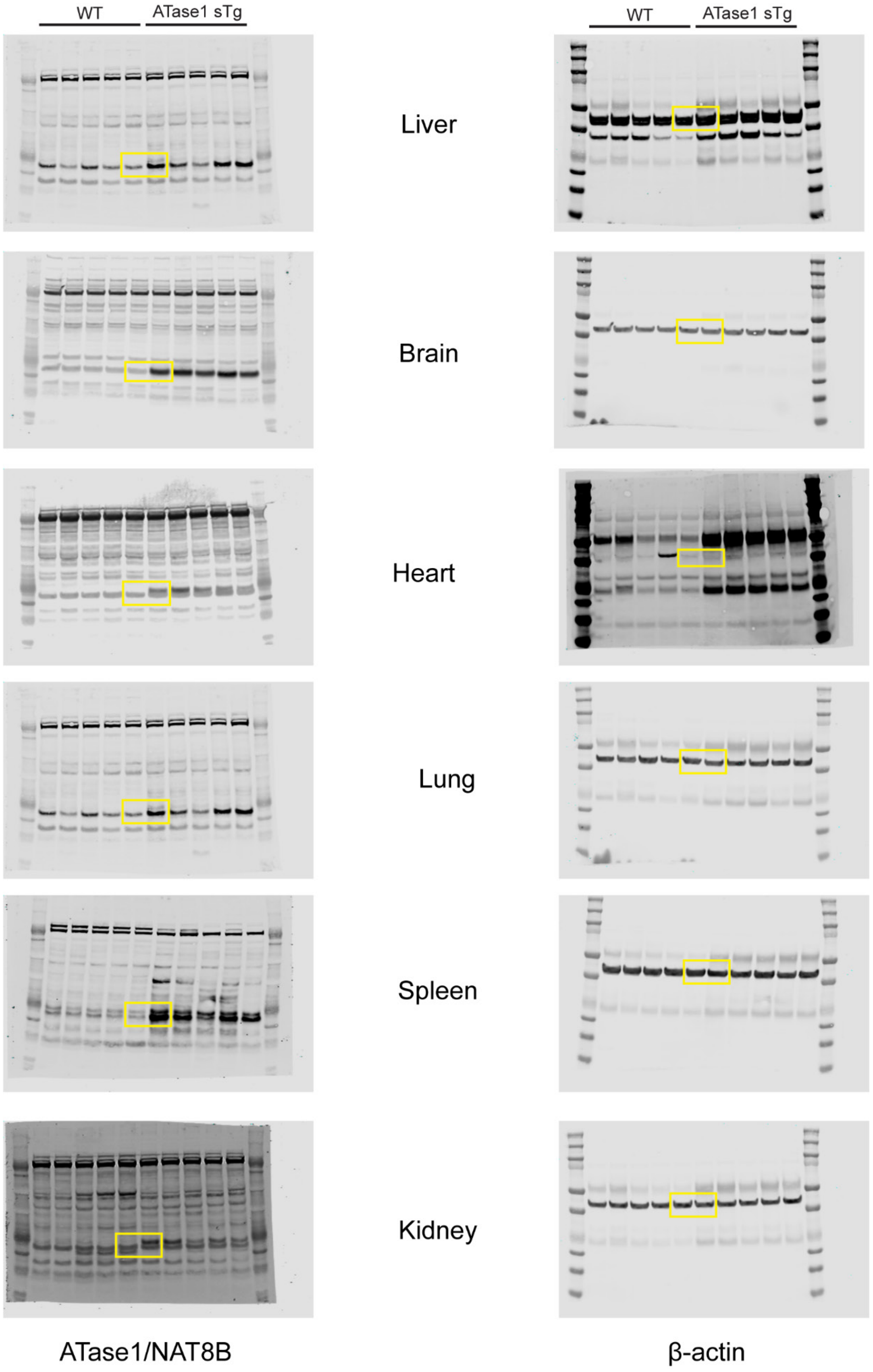
WT
ATase1 sTg
WT
ATase1 sTg
Liver
Brain
Heart
Lung
Spleen
Kidney
ATase1/NAT8B
β-actin
