# Supplementary figures and images for: Overexpression of ATase1 and ATase2 disrupts the secretome and causes a progeria phenotype
Source: Life Sci Alliance. 2025 Sep 10;8(12):e202503378. doi: 10.26508/lsa.202503378 (PMC12423556; doi:10.26508/lsa.202503378)

SOURCE DATA 2

Original Western blots from Figure 3

- 1. WT
- 2. ATase2 sTg

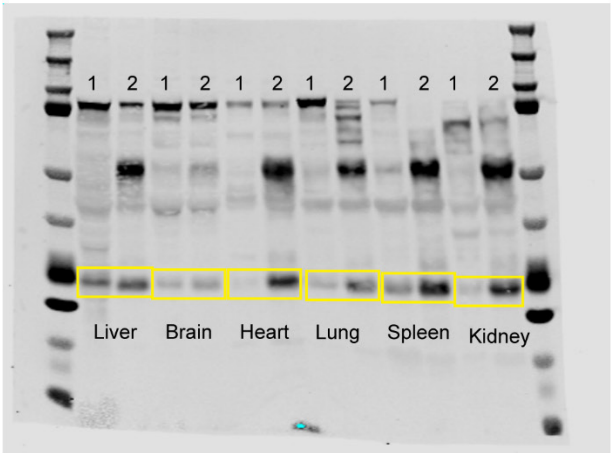

ATase2/NAT8

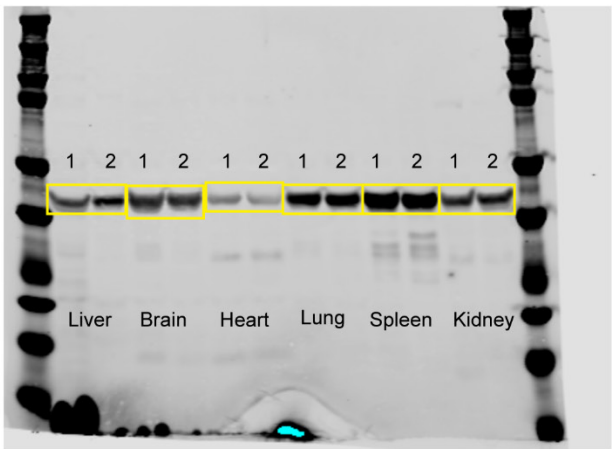

β-actin

Supplement: Supplementary file 3 [file LSA-2025-03378_SdataF3.1.pdf]

### **SOURCE DATA 3**

Original Western blots from Figure 8

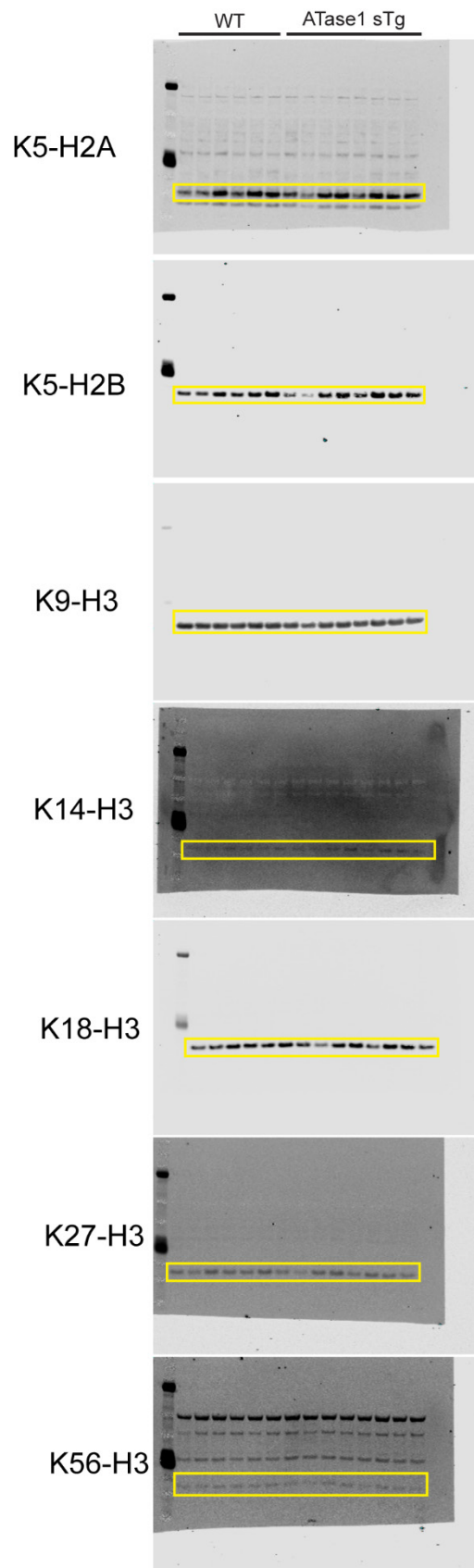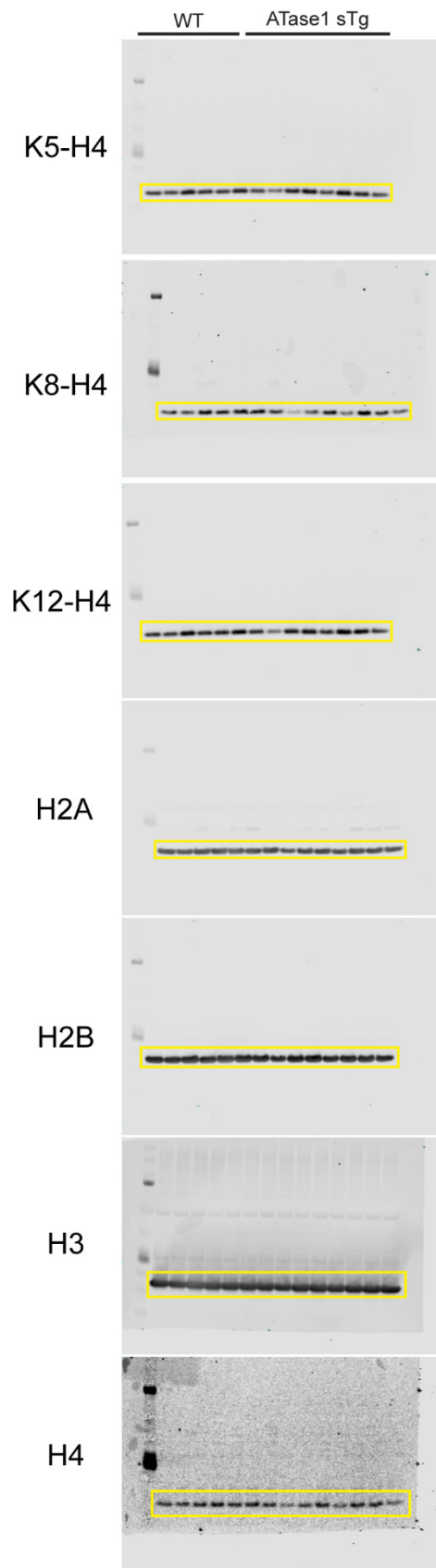

Supplement: Supplementary file 4 [file LSA-2025-03378_SdataF8.1.pdf]
